# P450 Fusion Protein Expressed in *E. coli* for Regioselective Hydroxylation of Flavonoids

**DOI:** 10.3390/molecules31122189

**Published:** 2026-06-22

**Authors:** Kinga Dulak, Agata Matera, Sandra Sordon, Maciej Wolak, Kinga Hyla, Ewa Huszcza, Jarosław Popłoński

**Affiliations:** 1 Department of Food Chemistry and Biocatalysis, Wroclaw University of Environmental and Life Sciences, 50-375 Wroclaw, Poland; kinga.dulak@upwr.edu.pl (K.D.); agata.matera@upwr.edu.pl (A.M.); sandra.sordon@upwr.edu.pl (S.S.); maciekwolak96@gmail.com (M.W.); ewa.huszcza@upwr.edu.pl (E.H.); 2 Department of Chemical Biology, Faculty of Biotechnology, University of Wrocław, Joliot-Curie 14a, 50-383 Wrocław, Poland; 3 Department of Biotechnology and Food Microbiology, Wroclaw University of Environmental and Life Sciences, 50-375 Wroclaw, Poland; kinga.hyla@upwr.edu.pl

**Keywords:** flavonoid hydroxylation, protein engineering, N-terminal modification, linkers design, P450, baicalein

## Abstract

Plant cytochrome P450 monooxygenases (CYPs) are valuable biocatalysts for the regioselective hydroxylation of aromatic compounds. However, their expression in bacterial hosts is hampered by poor solubility, membrane anchoring and the requirement for redox partners. In this work, we report the design and characterization of modular expression systems that enable the functional production of SbCYP82D1.1 from *Scutellaria baicalensis* (SbF6H) in *Escherichia coli*. Both independent expression and synthetic fusion systems were evaluated by combining a CYP with a compatible reductase (ATR2_tr from *Arabidopsis thaliana*) to catalyze the conversion of chrysin into baicalein. A combinatorial library of N-terminal variants, host strains, media, and induction strategies was constructed and screened. Among the tested host, *E. coli* DH 10-beta provided the highest product titers, particularly when cultures were supplemented with 5-aminolevulinic acid. Truncation of the native transmembrane anchor significantly improved catalytic performance, whereas the addition of the heterologous MALLLAVF tag decreased activity. Fusion systems outperformed separate expression formats, showing approximately two-fold higher activity, with the flexible glycine–serine linker (L_GS) supporting the highest hydroxylation product formation. The corresponding fusion construct showed an apparent conversion of 0.1 mM chrysin to baicalein of up to 90% under the applied whole-cell reaction and analytical conditions, although this value should be interpreted with caution due to the concurrent instability of baicalein observed in all reactions and culture conditions. This result nevertheless indicates a marked improvement in whole-cell baicalein formation compared with previously reported bacterial systems. Together, these results demonstrate that rational N-terminal engineering combined with fusion protein design can enable efficient bacterial expression of plant CYPs, representing a promising step toward scalable production of hydroxylated flavonoids.

## 1. Introduction

Cytochrome P450 enzymes are found in all domains of life and represent one of the most extensive and diverse protein families recognized to date [1]. Their catalytic versatility underpins a range of crucial physiological and biochemical processes [2]. Given their catalytic potential, particularly in the synthesis of pharmaceuticals, vitamins, flavors, fragrances, and agrochemicals, P450 enzymes remain a focal point of biotechnological interest [3]. However, efficient P450 recombinant expression remains challenging due to the necessity for correct heme incorporation, the requirement of a compatible redox partner, and the fact that most of P450s are membrane-associated [4].

*Escherichia coli* remains the expression host of choice due to its well-characterized genetics, fast growth, and ease of manipulation. Nevertheless, it lacks the machinery for many eukaryotic post-translational modifications and limited adaptation for the expression of membrane-bound proteins. Various strategies have therefore been implemented to improve the heterologous expression of P450s in *E. coli* [5,6]. Among these, the modification of the N-terminal domain, particularly through the substitution of hydrophobic signal N-terminal sequences that may act as nucleation factors and induce inclusion body formation, has shown significant promise [7]. The use of heterologous N-terminal sequences, such as MALLLAVF from bovine P450, has been widely adopted to enhance solubility and expression [8,9,10,11], though alternative sequences have also been explored [12]. N-terminal truncation is another effective approach [7], albeit with the caveat that it may impair enzymatic activity in some cases [13]. Additional strategies include co-expression with cytochrome P450 reductase (CPR) [9], CPR combined with cytochrome b5 [14], or molecular chaperones [10]. Optimizing culture conditions also contributes significantly to improved expression yields [15].

Recombinant fusion proteins consist of functional domains connected by a linker, which is essential for proper folding, expression, and catalytic activity [16]. Linker properties such as length, hydrophobicity, and amino acid composition influence domain orientation and structural integrity [17]. A notable natural example is cytochrome P450 BM3 from *Bacillus megaterium*, a fusion of fatty acid hydroxylase with its reductase partner exhibiting the highest known turnover rate (k_cat_) among P450s enzymes [18]. Numerous CYP fusion proteins with redox partners have been reported, including CYP51–ferredoxin fusion [19], members of the self-sufficient CYP505 subfamily containing an integral reductase domain [20], and CYP116B234 fused to its phthalate-family oxygenase reductase (PFOR) domain [21]. Engineered constructs have also been reported, such as P450 BM3 fused with formate dehydrogenase [22], carotene cyclase fused with hydroxylase [23], and ferredoxin reductase–ferredoxin applied with P450 monooxygenase [24]. Moreover, naturally occurring fusions have been identified that do not necessarily function in electron transfer [25], such as the STORR, which combines a P450 and CPR domain, where the CPR catalyzes the conversion of 1,2-dihydroreticulin to (R)-reticulin [26].

Flavonoids, a secondary plant metabolite, exhibit several health-promoting properties attributed to the presence of hydroxyl groups, particularly the catechol motif, which determined effective free radical scavenging, inhibition of lipid peroxidation, and metal ion complexation [27]. A key structural modification enabling catechol formation is the regioselective hydroxylation of the flavonoid ring [28]. However, despite notable progress, the selective and efficient hydroxylation of aromatic compounds using classical synthetic methods remains challenging, with current approaches offering limited regioselectivity [29]. On the other hand, the broad potential of monooxygenases described in the literature has not translated effectively into practical applications involving microorganisms or isolated enzymes as biocatalysts. Bacterial Rieske-type oxygenases contain an oxygen-sensitive [2Fe-2S] cluster [30], copper oxidases show low selectivity [31], and flavoprotein monooxygenases, key enzymes in catechol formation, remain poorly characterized, limiting their industrial applicability [32]. Most monooxygenases characterized to date belong to the cytochrome P450 family, which requires a compatible reductase for catalytic activity [3]. SbF6H (CYP82D1.1) from *Scutellaria baicalensis* [33] and GmF6H (CYP71D9) from *Glycine max* [34], when expressed in eukaryotic systems, demonstrated the capacity to hydroxylate flavonoids at the C-6 position. Expression of SbF6H in *E. coli* enabled the production of baicalein from phenylalanine with a conversion of 1.7% [35].

Given the extremely low rates of their catalyzed reactions and the growing interest in application of cytochrome P450 monooxygenases, the development of an efficient enzymatic electron transport system using recombinant proteins may represent a promising strategy to increase the hydroxylation yield and broaden the scope of P450 protein engineering. Furthermore, monooxygenase-based biocatalysis offers a sustainable and selective alternative for aromatic hydroxylation. The development of robust biocatalytic systems for synthesizing compounds relevant to the cosmetic and pharmaceutical industries remains a high priority. In this context, the present study explores two distinct strategies for expressing the biocatalytically active P450 system: monocistronic co-expression of CYP and CPR enzymes and single polypeptide chain fusion of CYP and CPR.

## 2. Results

### 2.1. Amino Acid Sequence Analysis

Bioinformatic characterization of the cytochrome P450 enzymes (SbF6H, GmF6H) and cytochrome P450 reductase (ATR2_tr) was performed using the HMMER web server (phmmer search|HMMER (ebi.ac.uk)), SMART domain annotation (SMART: Main page (embl.de)), and TMHMM-2.0 for transmembrane helix prediction (TMHMM 2.0—DTU Health Tech—Bioinformatic Services). Based on the output, transmembrane helices were identified for SbF6H (residues 5-24 aa) and GmF6H (residues 4-22 aa) (Supplementary Figure S1). For ATR2_tr, a previously described truncated variant lacking membrane association was used [36]. To generate possibly more soluble versions of SbF6H and GmF6H, the identified N-terminal membrane-bound segments were removed via PCR amplification and cloning of truncated variants.

### 2.2. Media Optimalization and Induction Strategy

**Figure 1:**
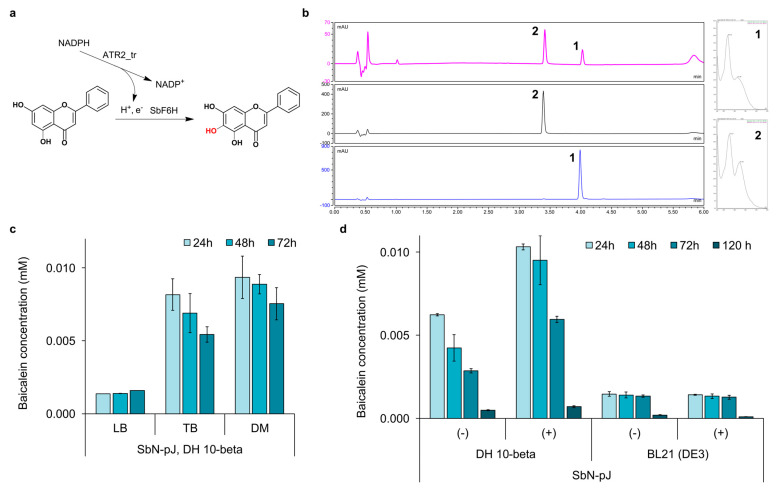
(**a**) Scheme of the hydroxylation reaction of chrysin by SbF6H. (**b**) UPLC analysis of the chrysin standard, baicalein standard, and reaction sample obtained after in vivo reaction (pink line—in vivo reaction, black line—standard of baicalein, blue line—standard of chrysin). The product peak was assigned to baicalein based on comparison with the authentic standard and supported by UV–Vis and LC-MS/MS data. UPLC profiles were determined by a photodiode array detector. 1: chrysin, 2: baicalein. (**c**) In vivo formation of baicalein by the SbN-pJ strain in different media (LB, TB, and DM) and (**d**) with and without the 5-ALA supplementation in DM. Chrysin was used as a substrate at an initial concentration of 0.1 mM. Data in all panels represent averages over three replicates. Error bars are not visible when smaller than the bar outline. Abbreviations: pJ—promoter pJ23100, SbN-pJ—N-truncation Sb6H variant, LB—Lysogeny Broth, TB—Terrific Broth, DM—Dynamite Medium, 5-ALA—5-aminolevulinic acid.

To optimize the co-expression of the CYPs and CPR, three media formulations, Lysogeny Broth (LB), Terrific Broth (TB), and Dynamite Medium (DM), were tested using SbN-pJ as a model construct. Sb6H co-expressed with ATR2_tr catalyzed conversion of chrysin to baicalein (Figure 1a,b). In vivo baicalein production was the lowest in LB, while both TB and DM supported markedly higher bioconversion efficiency, with chrysin-to-baicalein conversion approximately 8- and 10-fold greater, respectively (Figure 1c).

The impact on enzyme activity of 5-aminolevulinic acid (5-ALA) supplementation, a porphyrin biosynthesis precursor, was further assessed. The SbN-pJ construct was expressed in *E. coli* DH 10-beta and BL21 (DE3), with and without 5-ALA supplementation. A marked increase (>40%) in baicalein formation was observed in DH 10-beta with 5-ALA (Figure 1d), while in BL21 (DE3), product levels were negligible and unaffected by 5-ALA. Notably, baicalein levels declined over time in both strains, indicating possible product instability and degradation.

### 2.3. Induced Bacterial Growth Assay

To facilitate a smoother workflow for in vivo hydroxylation, the effect on *E. coli* growth of two key supplements, 5-aminolevulinic acid and rhamnose (Rhm, for rhamnose-inducible vectors), was evaluated. The tested strains carried plasmids encoding CYPs and CPR, either as monocistronic constructs or fusion proteins under constitutive or rhamnose-inducible promoters. Growth curves were recorded for cultures cultivated in mixed medium, with or without additional supplementation. No significant differences in growth kinetics or final cell density were observed between the supplemented and non-supplemented cultures (Supplementary Figure S2). The only notable exception was the strain harboring the GmN-pJ plasmid, which exhibited approximately a 50% reduction in optical density after 24 h of cultivation. Based on these observations, all subsequent experiments were conducted using the medium mix supplemented with 5-ALA and rhamnose (when applicable) added at the start of cultivation.

### 2.4. N-Terminal Library Screening

**Figure 2:**
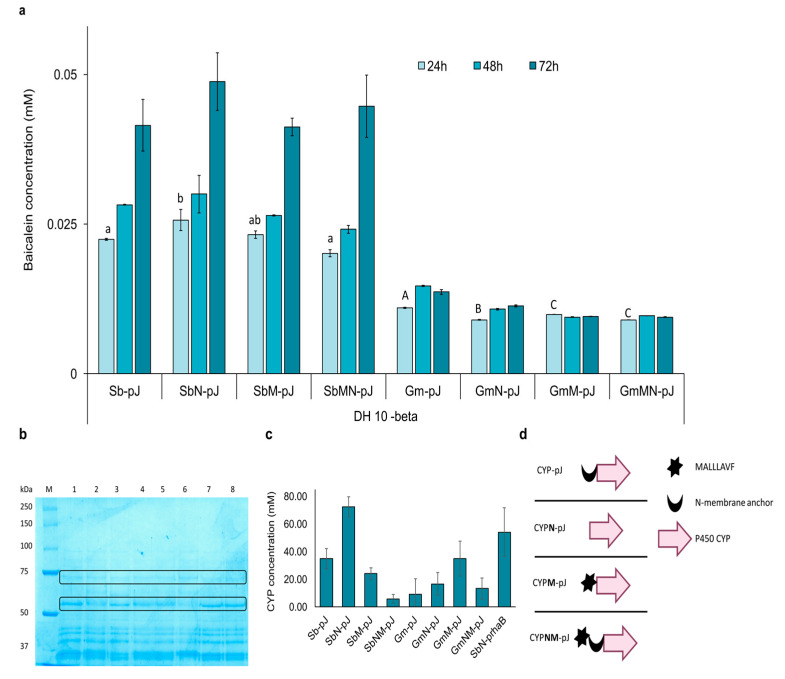
(**a**) In vivo baicalein formation by different N-terminal variants of SbF6H (Sb-pJ, SbN-pJ, SbM-pJ, SbNM-pJ) and GmF6H (Gm-pJ, GmN-pJ, GmM-pJ, GmNM-pJ) in Dynamite Medium. Chrysin was used as a substrate at an initial concentration of 0.1 mM. Data are presented as mean ± SD, n = 3 independent experiments. Error bars are not visible when smaller than the bar outline. Statistical significance was assessed using one-way ANOVA followed by Tukey’s multiple comparison test (SbF6H: F = 7.904, *p* = 0.009, GmF6H: F = 72.25, *p* = 0.000). F and *p* values are provided in the Supplementary Table S5. Different letters indicate statistically significant differences between different N-terminal variants regarding activity of SbF6H and GmF6H after 24 h of in vivo reaction (*p* < 0.05). (**b**) SDS-PAGE gel electrophoresis of crude extract containing CYP-CPR enzyme pairs co-expressed from different N-terminal variant plasmids. The frames highlight the enzyme fraction of the hydroxylase SbF6H, GmF6H, and reductase ATR2_tr, ladder (M), Sb-pJ (line 1), SbN-pJ (line 2), SbM-pJ (line 3), SbMN-pJ (line 4), Gm-pJ (line 5), GmN-pJ (line 6), GmM-pJ (line 7), GmNM-pJ (line 8). Expected mass of recombinant protein: Sb6H—58.6 kDa, Sb6H_trN—56.00 kDa, Gm6H—56.2 kDa, Gm6H_trN—53.6 kDa, ATR2_tr—71.2 kDa. (**c**) CYP concentration in different N-terminal variants of SbF6H and GmF6H. (**d**) Scheme of a library of different N-terminal constructs. Abbreviations: pJ—promoter pJ23100, prhaB—promoter rhaBAD.

Following optimization of cultivation conditions and the supplementation strategy, we screened a library of N-terminal variants of SbF6H and GmF6H (Figure 2d). The goal was to improve catalytic performance by modifying or removing membrane-associated segments. Each variant was cloned into the pSEVA63g19gA vector [37] under the constitutive promoters (pJ23100 for CYPs, pJ23102 for ATR2_tr) (Table 1, Supplementary Table S1, Transcriptional Unit section, Supplementary Table S2). The library included four variants per enzyme, generated through truncations and sequence modifications. All constructs were introduced into the DH 10-beta *E. coli* strain and tested for in vivo hydroxylation of chrysin over 72 h (Figure 2a). Baicalein production confirmed catalytic activity in all variants. Interestingly, relative to previous assays, baicalein concentration was almost five times higher, indicating a positive effect of supplementation from the start of the culture on enzyme activity. Nevertheless, GmF6H variants showed consistently low product formation, especially the N-MAL-tagged strain, with no increase over time. In contrast, SbF6H variants exhibited gradual baicalein accumulation, with the truncated SbN-pJ variant (no tag) demonstrating the highest activity. This suggests that membrane anchoring fragment removal slightly enhances function, whereas fusion with a MALLLAVF tag did not have any significant effect. SDS-PAGE confirmed CYP and CPR expression in all constructs (Figure 2b). The highest cytochrome P450 (CYP) concentrations, as determined by CO-difference spectroscopy, were observed for SbF6H in the construct lacking the N-terminal fragment (SbN-pJ) and for GmF6H containing the N-terminal hydrophilic MALLLAVF extension (GmM-pJ) (Figure 2c). Based on activity profiles and CYP expression levels, SbN-pJ was selected for further experiments.

### 2.5. Host Strains Evaluation for CYP Production

**Figure 3:**
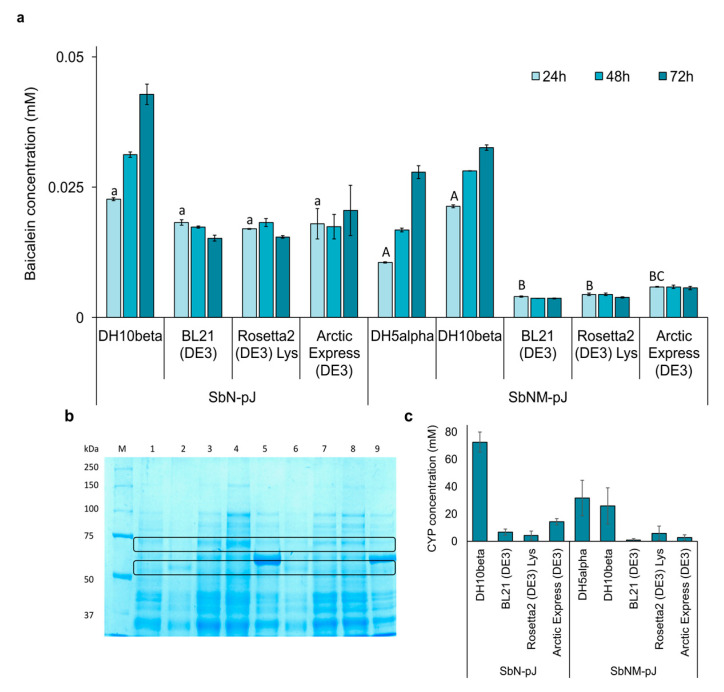
(**a**) In vivo baicalein formation by two N-terminally modified SbF6H variants (SbN-pJ, SbNM-pJ) in different *E. coli* host strains in Dynamite Medium. Chrysin was used as a substrate at an initial concentration of 0.1 mM. Data are presented as mean ± SD, n = 3 independent experiments. Error bars are not visible when smaller than the bar outline. Statistical significance was assessed using one-way ANOVA followed by Tukey’s multiple comparison test (SbN-pJ: F = 2.182, *p* = 0.168, SbNM-pJ: F = 80.81, *p* = 0.000). F and *p* values are provided in Supplementary Table S6. Different letters indicate statistically significant differences between different *E. coli* host strains after 24 h of in vivo reaction (*p* < 0.05). (**b**) SDS-PAGE gel electrophoresis of crude extract containing the CYP-CPR enzyme pairs co-expressed in various *E. coli* strains. The frames highlight the enzyme fraction of the hydroxylase SbF6H and reductase ATR2_tr, ladder (M), SbN-pJ: DH 10-alpha (line 1), DH 10 beta (line 2), BL21 (DE3) (line 3), Rosetta2 (DE3) pLysS (line 4), Arctic Express (DE3) (line 5), SbMN-pJ: DH 10-beta (line 6), BL21 (DE3) (line 7), Rosetta2 (DE3) pLysS (line 8), Arctic Express (DE3) (line 9). Expected mass of recombinant protein: Sb6H_trN—56.00 kDa and ATR2_tr—71.2 kDa. (**c**) CYP concentration expressed in different *E. coli* strains. Abbreviations: pJ—promoter pJ23100, SbN-pJ—N-truncation Sb6H variant, SbNM-pJ—N-truncation SbF6H variant with added MALLLAVF sequence.

To assess the effect of host on enzyme performance, the two top SbF6H variants (SbN-pJ and SbMN-pJ) were expressed in five *E. coli* strains: DH 5-alpha, DH 10-beta, BL21 (DE3), Rosetta2 (DE3) pLysS and Arctic Express (DE3). Product formation varied strongly with host type (Figure 3a). The highest baicalein production was observed in *E. coli* DH 5-alpha (SbNM-pJ) and DH 10-beta (SbN-pJ, SbNM-pJ). This correlated with detectable CPR expression in all strains and a clear CYP signal especially in DH 10-beta, based on SDS-PAGE analysis (Figure 3b), as well as the highest CYP concentrations in these two (DH 5-alpha, DH 10-beta) bacterial strains (Figure 3c). In BL21 (DE3), a strain commonly employed for recombinant protein production, enzyme activity was significantly reduced, and no detectable bands were observed for CYP. Similarly, Rosetta2 (DE3) pLysS and Arctic Express (DE3) displayed negligible enzyme activity and low expression levels. Despite the generally low detectability of CYPs by SDS-PAGE across strains, baicalein production confirmed that the enzymes were produced and at least a fraction is catalytically active. Unlike in earlier experiments, a consistent increase in baicalein levels over time was observed. For further experiments, *E. coli* DH 10-beta was chosen as the model host for in vivo hydroxylation.

### 2.6. Fusion Protein Assay

**Figure 4:**
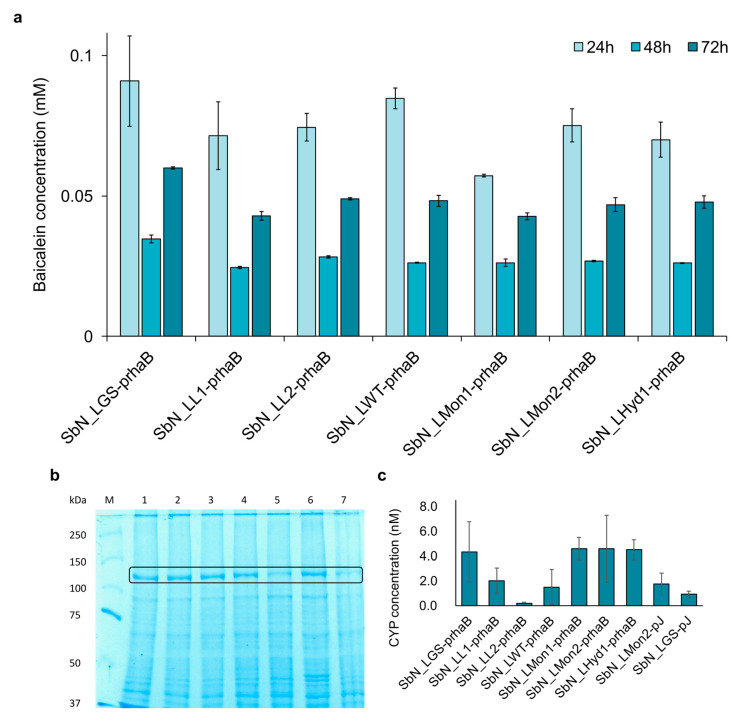
(**a**) In vivo baicalein formation by SbF6H_trN-ATR2_tr fusion proteins linked by different linkers expressed in the DH 10-beta *E. coli* strain in Dynamite Medium. Chrysin was used as a substrate at an initial concentration of 0.1 mM. Data are presented as mean ± SD, n = 3 independent experiments. Error bars are not visible when smaller than the bar outline. Statistical significance was assessed using one-way ANOVA followed by Tukey’s multiple comparison test. No statistically significant differences were observed between the linker variants after 24 h of in vivo reaction (F = 0.43, *p* = 0.847). Detailed ANOVA and post hoc results are provided in Supplementary Table S7. (**b**) SDS-PAGE gel electrophoresis of crude extracts containing CYP-CPR enzyme pairs co-expressed as fusion protein linked by different linkers. The frames highlight the enzyme fraction CYP-CPR fusion protein, ladder (M), SbN_LGS-prhaB (line 1), SbN_LMon1-prhaB (line 2), SbN_LMon2-prhaB (line 3), SbN_LHyd1-prhaB (line 4), SbN_LL1-prhaB (line 5), SbN_LL2-prhaB (line 6), SbN_LWT-prhaB (line 7). Expected mass of recombinant protein: Sb6H_trN and ATR_tr genes fused with linker: L_GS—128.5 kDa, L_Mon1—128.4 kDa, L-Mon2—129.5 kDa, L_Hyd1—128.5 kD, L_L1—128.5 kDa, L_L2—129.1 kDa, L_WT—129.6 kDa. (**c**) CYP concentration in fusion proteins containing different linker types. Abbreviations: pJ—promoter pJ23100, prhaB—promoter rhaBAD.

Modular combinatorial cloning was performed using a set of nine linkers (Table 2) with and without the MALLLAVF tag. Following transformation to *E. coli* DH 10-beta and double selection on antibiotic and X-gal plates, 50 colonies were selected. Based on blue-white screening, 40 positive clones were tested for the in vivo chrysin hydroxylation assays. No baicalein production was observed in MALLLAVF tagged constructs, suggesting that the presence of this N-tag sequence interfered with fusion enzyme function or expression. Among the untagged variants, 29 and 39 clones showed product formation after 24 and 48 h, respectively (Supplementary Figure S3). Based on the baicalein conversion rates, the clones were categorized into six groups: >15%, 10–15%, 8–10%, 5–8%, 2–5%, and 0.1–2%. While most clones demonstrated a decrease in product levels over time, an opposite trend was observed in the lowest-activity group (0.1–2%), where baicalein production increased between 24 and 48 h. From the full dataset, 20 representative clones, at least three from each performance category, were selected for sequencing and further analysis. Variants containing monooxygenase-specific linkers, SbN_LMon1-prhaB, SbN_LMon2-prhaB and SbN_LHyd1-prhaB, exhibited improved activity compared to the monocistronic reference system (SbN-pJ). However, despite having one of the highest CYP concentrations, these constructs ranked among the least effective within the fusion protein library (Figure 4a,c). Nonetheless, all three designs outperformed the separate production of CYP and CPR, confirming the functional advantage of the fusion strategy. The highest baicalein production was achieved using the SbN_LGS-prhaB linker, a widely used flexible glycine–serine motif, which correlated with the high CYP concentration obtained for this construct. Its modified versions (SbN_LL1-prhaB, SbN_LL2-prhaB, and SbN_LWT-prhaB) also supported product formation, highlighting their compatibility and effectiveness in the fusion protein context. In all functional constructs, baicalein levels peaked at 24 h, followed by a consistent decline after 48 and a subsequent increase at 72 h. This trend suggests instability of the product over prolonged incubation. To investigate this discrepancy and assess the compound’s stability, additional time-course experiments were performed, as described in next section.

SDS-PAGE analysis confirmed visible expression of the full-length fusion protein in all tested constructs (Figure 4b), demonstrating the ability of *E. coli* to express large proteins containing multiple enzymatic domains and supporting the chosen approach. Western blot analysis using anti-His antibodies was also performed and revealed that His-tagged protein bands were barely detectable for most constructs, whereas strong signals were observed for SbN_LL1-prhaB and SbN_LL2-prhaB (Supplementary Figure S4). Notably, no signal was detected for the SbN_LWT-prhaB construct, which was consistent with its markedly lower band intensity observed in the SDS-PAGE analysis. It should be also noted that the protein abundance estimated by SDS–PAGE and Western blot analyses does not necessarily reflect the concentration of the catalytically active CYP enzyme. Both methods were used primarily to confirm the production of the CYP–CPR fusion proteins (~130 kDa) and detect protein present in the samples. In contrast, CO difference spectroscopy specifically detects properly folded, heme-incorporated CYP proteins that are catalytically competent. Consequently, discrepancies between electrophoretic protein signals and catalytic activity may arise from differences in the proportion of functional enzymes. A fraction of the expressed fusion protein may be misfolded, improperly assembled, or lack heme incorporation and therefore contribute to the SDS–PAGE or Western blot signal while remaining catalytically inactive. Furthermore, Western blot signal intensity may be influenced by factors such as transfer efficiency and epitope accessibility and should not be considered as a direct measure of protein abundance. Therefore, catalytic activity is expected to correlate more closely with the concentration of functional CYP determined by CO difference spectroscopy than with the total protein levels estimated by electrophoretic methods.

### 2.7. Baicalein Stability in Culture Conditions

The ambiguity of the results, including the downward trend observed in the initial assays (Figure 1), the upward trend in subsequent assays (Figure 2 and Figure 3), and the fluctuations observed during verification of fusion protein activity (Figure 4), strongly suggests that the produced baicalein undergoes degradation during culture. To evaluate baicalein stability, degradation assays were conducted in cultures with and without CYP-CPR expression. In both cases, baicalein levels dropped dramatically within the first 6 h, with only 15% remaining by 48 h (Supplementary Figure S5a). Similar degradation observed in control cultures indicates that the product was degraded independently of the production of the tested enzymes. Stability testing with chrysin as a substrate showed that baicalein levels increased over the first 8 h, remained stable until 24 h, and then progressively declined, indicating product degradation over time (Supplementary Figure S5b). These results underscore the need to consider product stability when evaluating bioconversion efficiency. Nonetheless, despite inconsistencies in the results caused by baicalein degradation, we proceeded with the same analytical and similar reaction setups for the sake of possible comparison of the expression systems.

### 2.8. Comparison of Expression Formats: Monocistronic vs. Fusion

**Figure 5:**
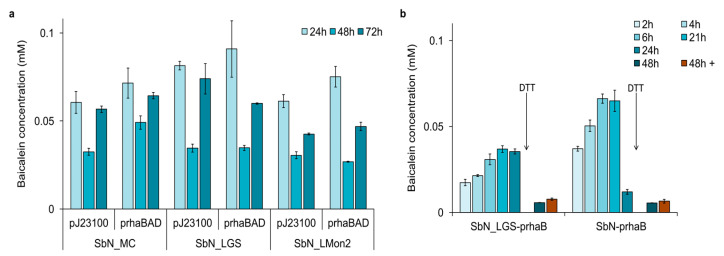
(**a**) In vivo baicalein formation by SbF6H_trN and ATR2_tr produced by different expression systems in the DH 10-beta *E. coli* strain. (**b**) Baicalein formation by SbN_LGS-prhaB and SbN-prhaB in resting-cell reactions. DTT (25 mM) was added to half of the cultures after 24 h. Chrysin was used as a substrate at an initial concentration of 0.1 mM. Data in all panels represent averages over three replicates. Error bars are not visible when smaller than the bar outline. Abbreviations: MC—monocistronic constructs.

To identify the most effective system for baicalein production, we compare the best-performing N-terminal variant with two CYP-CPR fusion proteins: one using the SbN (the best N-terminal variant), another using the SbN_LGS (the top-performing system overall) and the other using SbN_LMon2 (the best linker among those tailored for monooxygenases). Each was expressed under constitutive and rhamnose-inducible promoters (Figure 5a). Rhamnose induction improved product formation in all systems. The L_GS-based fusion construct showed the highest overall activity. As in earlier tests, baicalein levels fluctuated over time, confirming instability of the product in the culture environment.

In our previous studies on C-8 hydroxylation of flavonoids with FAD-dependent monooxygenases, we have also noticed similar product and substrate instability in microbial cultures. Back then, supplementation of reducing agents in in vitro reactions greatly lowered product degradation [38]. The addition of a strong reducing agent (DTT) to the culture medium negatively affected the growth of cells carrying an additional plasmid, as well as on those without it (Supplementary Figure S6). Switching to resting cells resulted in an almost two-fold decrease in yield compared to growing cells (Figure 5). In the context of baicalin degradation, no increase in baicalin concentration was observed between 6 and 21 h, suggesting that extending the reaction beyond 6 h is not justified. Moreover, a dramatic decrease in its concentration was observed for the SbN-prhaB strain between 21 and 24 h of the reaction (Figure 5b). After 24 h, 25 mM DTT was added to half of the culture of both strains (SbN_LGS-prhaB and SbN-prhaB). However, the results obtained after 48 h indicate only a slight improvement in baicalin stability in this experiment, which might suggest that the redox potential of the medium is not the main factor of degradation.

### 2.9. Substrate Scope

We also analyzed the catalytic potential of the enzyme SbF6H using flavonoids and steroids as substrates in in vivo reactions. The enzyme exhibited activity against naringenin, chrysin, apigenin, luteolin, kaempferol and isoxanthohumol (Supplementary Table S3, Supplementary Figures S7–S12). These results considerably broaden the known substrate specificity of the enzyme. However, these assays necessitate further investigation, particularly to determine the structure of the resulting products and to identify the specific hydroxylation sites, although the UV–Vis spectra of the products observed align with those in the literature for corresponding C-6 hydroxylation and MS/MS fragmentation patterns support ring A hydroxylation. However, detailed analysis of reaction products falls beyond the scope of this study.

## 3. Discussion

This study explores the role of N-terminal engineering and fusion protein design in optimizing the heterologous expression and catalytic activity of plant-derived cytochrome P450 monooxygenases in *E. coli*. Removal of hydrophobic transmembrane anchors, particularly in the case of SbF6H, led to a substantial enhancement in the enzyme activity. This observation aligns with previous findings indicating that modification of N-terminal membrane regions can reduce the formation of inclusion bodies and improve protein folding efficiency [7]. Expression of several eukaryotic P450 enzymes has been markedly improved by replacing their native N-terminal regions with the LLLAVFL sequence [8,9,10,11]. For example, in human CYP1A2, this substitution increased protein yields from less than 2 nmol/L to 225 nmol/L of culture [11]. Another commonly employed N-terminal tag is the hydrophilic sequence KKTSSKGR, which, while not affecting membrane association, has been reported to promote even greater expression levels that the LLLAVFL motif [39]. However, the widespread application of N-terminal tags negatively affected the enzymatic activity of the tested proteins, especially in the case of SbF6H, where the truncated, untagged variant (SbN-pJ) outperformed the tagged versions. Sequence modification based solely on truncation of the N-terminal membrane anchor has been applied to numerous CYPs expressed in bacterial systems [6,7,11,12]. Nevertheless, due to the variability in outcomes, such as the enhanced expression of CYP3A37 [40] versus the negligible effect observed for CYP2B6 [41], the impact of individual truncations remains difficult to predict. These findings suggest that, while N-terminal truncation and heterologous tagging can enhance solubility and expression, their effects are enzyme-specific and need to be confirmed empirically.

The choice of *E. coli* host strain is another key factor influencing recombinant P450 expression and catalytic efficiency. The DH 5-alpha strain is frequently employed for the production of recombinant CYP proteins [42,43,44,45,46,47,48]. Expression in the JM109 strain is also commonly reported [48,49], although there are instances where enzymes were inefficiently expressed by JM109 but produced successfully in DH 5-alpha [48]. These findings are consistent with our results, in which DH 5-alpha and DH 10-beta emerged as the most effective co-expression hosts for CYP-CPR systems among the five tested strains. However, the literature also provides examples of successful CYP expression in other *E. coli* strains, including MV1304 [41], XL-1 blue [50], TOPP3 [51], and Rosetta (DE3) Lys2 [52]. Therefore, we recommend evaluating the expression capacity of multiple *E. coli* strains at the initial stages of a study involving the production of recombinant CYP enzymes.

Several factors influence the efficiency of CYP protein expression, including the choice of culture medium, induction temperature, duration of expression, and supplementation with 5-aminolevulinic acid (5-ALA). Among the tested media, the best results were obtained using Terrific Broth (TB) and Dynamite Medium (DM), which aligns with the frequent use of TB for CYP expression reported in the literature [15,53,54]. To enhance enzyme stability, TB is often supplemented with trace elements, such as FeCl_3_, MgCl_2_ and (NH_4_)_2_SO_4_ [44]. The DM, which expands TB composition with numerous inorganic salts, supported efficient co-expression of SbF6H_trN and ATR2_tr in our system. Furthermore, 5-ALA supplementation led to an almost two-fold increase in baicalein concentration compared to cultures lacking this additive. Although *E. coli* has an endogenous heme biosynthetic pathway sufficient for producing heme-containing proteins, supplementation with 5-ALA, a heme precursor, has been demonstrated to significantly enhance the expression level [55,56].

The simplification of the redox system required for CYP activity represents a great biotechnological advantage of fusion protein strategies [57,58]. Notable natural examples of such systems include BM3 from *B. megaterium* [18], CYP–flavodoxin and CYP–ferredoxin from *Methylococcus capsulatus* [59], and CYP116B from *Rhodococcus erythropolis* [60]. Numerous CYP-CPR fusion enzymes have been generated through genetic engineering [57,58]. However, a limitation of artificial fusion proteins is the rate of CYP reduction, which can restrict overall catalytic efficiency. For instance, fusion of rat fatty acid hydroxylase (CYP4A1) and bovine steroid hydroxylase (CYP17α) to rat CPR via a short Ser–Thr linker, with the N-terminal membrane anchor removed [61], did not improve the catalytic activity for the 17α-hydroxylation of progesterone or pregnenolone. However, a substantial enhancement in activity was observed for lauric acid hydroxylation upon addition of exogenous CPR to the CYP4A1-CPR fusion [62]. These findings underscore the importance of designing interdomain peptide linkers that support efficient electron transport between the CYP and CPR. Among the SbF6H_trN and ATR2_tr fusion tested constructs, the system employing the flexible L_GS linker demonstrated the highest activity. This linker has been widely used in the design of fusion proteins due to its beneficial effects on protein stability and folding [63,64,65], as well as its ability to enhance biological activity [63,66,67]. Other SbF6H_trN-ATR2_tr fusion constructs also supported catalytic activity comparable to or exceeding that observed with separately expressed enzymes. A comprehensive assessment of the effect of fusion system optimization on catalytic efficiency would require determination of the enzyme kinetic parameters. However, owing to the instability of the products in both in vivo and in vitro assays [38], together with the substantial overlap between the UV spectra of the flavonoids and the cofactor, reliable kinetic measurements are currently beyond the scope of analytical methods. Furthermore, the variability in catalytic activity observed between constructs may not be solely explained by the linker design or N-terminal modifications. Other factors should also be considered. A major bottleneck in CYP systems is often the limited intracellular availability of cofactors such as NAD(P)H and heme, which directly limits the catalytic turnover of both separate and fused enzymes [68,69]. Another important aspect is the metabolic burden caused by the expression of large fusion proteins, which can disrupt translation capacity, folding efficiency, and cellular redox balance, thereby lowering overall productivity [7]. In addition, insufficient electron transfer (uncoupling) may result in wasteful NADPH consumption and reactive oxygen species generation [68,70]. Although these factors were not addressed in this study, they represent key variables to be considered in the future optimization of bacterial CYP-CPR systems. Consequently, the increased whole-cell bioconversion efficiency observed in this study should be attributed to the combined effects of improved fusion architecture and expression characteristics rather than exclusively to changes in the intrinsic catalytic properties of the enzymes. According to Zhao et al., expression of SbF6H in yeast enabled the conversion of more than 50% of chrysin supplied to the culture at the initial substrate concentration of 5 µM. In vitro assays further confirmed the enzyme’s ability to hydroxylate other flavonoids, including pinocembrin, apigenin, and 7-*O*-methylchrysin. Based on these results, the authors suggested that SbF6H exhibits substrate promiscuity, capable of converting a range of flavones to their corresponding 6-hydroxylated derivatives with comparable efficiency [33]. However, expression of Sb6H in *E. coli* supplemented with 500 mg/L phenylalanine enabled the baicalein *de novo* production, yielding 8.5 mg/L with a 1.7% conversion rate [35]. The present study describes the first attempt to express the F6H hydroxylase from *S. baicalensis* in a fusion system with ATR2_tr from *A. thaliana* in a bacterial host. Under the applied whole-cell reaction and analytical conditions, the best-performing strain showed apparent conversion corresponding to 90.1% of the supplied chrysin (0.1 mM). However, because baicalein degradation occurred simultaneously with its formation, this value should not be interpreted as a full substrate–product mass balance or as a catalytic efficiency parameter. Nevertheless, the results demonstrate a substantial improvement in whole-cell baicalein formation compared with previously reported bacterial systems.

Despite the promising results, the presented study has certain limitations that should be considered when planning further work. The main issue is the instability of the reaction product, especially that it was not observed in previous studies on baicalein production. It has been shown that baicalein undergoes rapid degradation under bacterial culture conditions, irrespective of the presence of CYP-CPR systems. Therefore, we decided to modify the reaction environment based on our previous experience, which had enabled the production of 8-hydroxyquercetin [38]. Unfortunately, in this case, the applied strategy did not yield the expected results. DTT is gradually toxic for cells at a >3 mM concentration and is not metabolized by *E. coli*, thus acting as a reducing agent in the medium [71]. However, living cells with active metabolism diminish all the possible benefits of its application. Consequently, the potential biocatalytic application of the system requires further efforts aimed at improving compound stability. Due to baicalein degradation, no specific by-products were identified, and neither the mechanism nor the structure of the resulting metabolites were determined, which limits our understanding of the complete reaction pathway in *E. coli* cells. Nevertheless, there are reports indicating similar observations of flavonoid degradation [72,73,74,75] also related to microbial activity, and since not all *E. coli* proteins (including DH 5-alpha and DH 10-beta) have known and experimentally determined function, including promiscuous activity, there is a great chance of enzymatic activity towards both chrysin and baicalein. In particular, the NCBI database search within annotated sequences of these strains indicated proteins such as quercetinase *yhhW*, a homolog of an enzyme associated with flavonol degradation, were found in *Streptomyces* or *Bacillus subtilis* [76,77].

Another limitation arises from the relatively narrow substrate panel, which primarily included chrysin and only a few additional flavonoids. The limited substrate scope hinders a comprehensive evaluation of the enzyme’s engineering potential. Finally, further studies should include detailed kinetic analyses to enable a clear assessment of the impact of the linker design and N-terminal modifications on the catalytic parameters. In particular, all our efforts on the isolation and application of pure enzymes, both separately expressed and in fusion, led to no activity or only trace activity, limiting a real comparison of enzyme kinetic parameters.

## 4. Materials and Methods

### 4.1. Bioinformatics Assays

All DNA constructs were designed in silico using SnapGene software (version 5.1.7, Insightful Science, Boston, MA, USA). Protein sequence analyses were performed using HMMER (phmmer search|HMMER (ebi.ac.uk)) [78], SMART (SMART: Main page (embl.de)) [79], and TMHMM-2.0 (TMHMM 2.0—DTU Health Tech—Bioinformatic Services) [80] tools.

### 4.2. Bacterial Strains

*E. coli* strains NEB 5-alpha and NEB 10-beta (New England BioLabs, Ipswich, MA, USA) were employed for plasmid cloning and propagation. Reactions were conducted in *E. coli* NEB 5-alpha, NEB 10-beta, BL21 (DE3) (New England BioLabs, USA), Rosetta2 (DE3) pLysS (Merck MIllipore, Darmstadt, Germany) and Arctic Express (DE3) (Agilent Technologies, Santa Clara, CA, USA), depending on construct compatibility and observed activity.

### 4.3. DNA Manipulation

All oligonucleotides were synthesized by Merck Millipore (Germany) and are listed in Supplementary Table S1. Hybridization of complementary oligonucleotides was performed in a thermocycler using a gradient from 98 °C to 30 °C (stepwise decrease by 2 °C every 2 min), followed by phosphorylation with T4 Polynucleotide Kinase and ligation into the SmaI-digested and dephosphorylated (Quick CIP) pSEVA181 vector [81] using T4 DNA Ligase (all reagents from New England BioLabs, USA). Reactions were incubated overnight at room temperature.

Polymerase chain reactions (PCRs) were conducted in 50 μL volumes containing: 1 U Phusion Hot Start II Polymerase, 0.2 mM dNTPs, Phusion^®^ HF Reaction Buffer (Thermo Fisher Scientific, Waltham, MA, USA), 0.5 μM of each primer, and 50 ng of DNA template. PCR products were purified via 0.7% (*w*/*v*) agarose gel electrophoresis using a GeneJET Gel Extraction Kit (Thermo Fisher Scientific, USA). Purified PCR products were then mixed with the previously SmaI-digested pSEVA181 vector [81] in a 10:1 molar ratio, along with 1 μL T7 DNA Ligase and 1.5 μL T7 ligase buffer (New England Biolabs, USA), and incubated at room temperature overnight. DNA concentrations were determined using a Nanodrop spectrophotometer 2000 (Mettler Toledo, Warsaw, Poland). A total of 15 μL of the ligation mixture was transformed into chemically competent NEB 5-alpha cells, following the manufacturer’s heat shock protocol.

**Figure 6:**
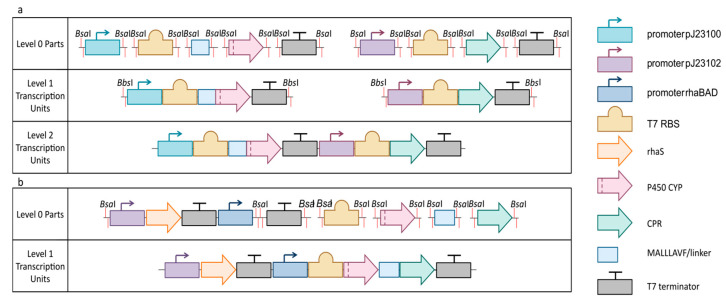
Modular assembly of transcription units using Golden Standard Technique (GS MoClo). Construction of genetic circuits from basic DNA parts (Level 0), including promoters, ribosome binding sites (RBSs), coding sequences (P450 CYP and CPR) and terminators. These parts were assembled into transcription units (Level 1), which were then combined into multigene constructs (Level 2) under (**a**) constitutive pJ23100 and pJ23102 promoters or (**b**) the rhamnose-inducible promoter. Dashed lines indicate the use of full-length or truncated CYP coding sequences. Abbreviations: P450 CYP—cytochrome P450 monooxygenase, CPR—reductase.

Construct assembly was performed using the Golden Standard Modular Cloning (GS MoClo) system [37]. CYP genes (SbCYP82D1.1 from *S. baicalensis* (SbF6H) [33], GmCYP71D9 from *G. max* (GmF6H) [34]) and *Arabidopsis thaliana* CPR without N-terminal membrane anchoring sequence (ATR2_tr)) [36,82] were codon-optimized and synthesized (Doulix, Venice, Italy). Two N-SbF6H variants (SbN-pJ and SbNM-pJ) and nine linkers (Table 2) were used in modular combinatorial cloning with and without the MAL tag (MALLLAVF) to construct fusion proteins. Constructs including all N-terminal and fusion variants were cloned into the pSEVA63g19gA vector [37] (J23100 promoter—CYP, pJ23102 promoter—CPR) and pRhaBAD_12 vector [83] (rhamnose-inducible promoter). All genetic parts were flanked by BsaI restriction sites for hierarchical assembly. Details of genes, oligonucleotides, vectors, and bacterial strains can be found in Figure 6 and Table 1 and Supplementary Materials Table S1. Transformations were carried out using chemically competent NEB 5-alpha and NEB 10-beta cells, following the manufacturer’s heat shock protocol.

**Table 1:** Genetic constructs used in this study.

ID	N-Modification	Linker	CDS	Expression Control
*N*-terminal library				
Sb-pJ	-	-	SbF6H, ATR2_tr	constitutive
SbN-pJ	N-truncation	-	SbF6H, ATR2_tr	constitutive
SbN-prhaB	N-truncation	-	SbF6H, ATR2_tr	rhamnose-inducible
SbM-pJ	N-MALLLAVF	-	SbF6H, ATR2_tr	constitutive
SbNM-pJ	N-truncation, MALLAVF	-	SbF6H, ATR2_tr	constitutive
Gm-pJ	-	-	GmF6H, ATR2_tr	constitutive
GmN-pJ	N-truncation	-	GmF6H, ATR2_tr	constitutive
GmM-pJ	N-MALLLAVF	-	GmF6H, ATR2_tr	constitutive
GmNM-pJ	N-truncation, MALLAVF	-	GmF6H, ATR2_tr	constitutive
Fusion protein				
SbN_LGS-pJ	N-truncation	L_GS	SbF6H, ATR2_tr	constitutive
SbN_LMon2-pJ	N-truncation	L_Mon2	SbF6H, ATR2_tr	constitutive
SbN_LGS-prhaB	N-truncation	L_GS	SbF6H, ATR2_tr	rhamnose-inducible
SbN_LL1-prhaB	N-truncation	L_L1	SbF6H, ATR2_tr	rhamnose-inducible
SbN_LL2-prhaB	N-truncation	L_L2	SbF6H, ATR2_tr	rhamnose-inducible
SbN_LWT-prhaB	N-truncation	L_WT	SbF6H, ATR2_tr	rhamnose-inducible
SbN_LMon1-prhaB	N-truncation	L_Mon1	SbF6H, ATR2_tr	rhamnose-inducible
SbN_LMon2-prhaB	N-truncation	L_Mon2	SbF6H, ATR2_tr	rhamnose-inducible
SbN_LHyd1-prhaB	N-truncation	L_Hyd1	SbF6H, ATR2_tr	rhamnose-inducible

### 4.4. Linker Design for Fusion Constructs

To create the CYP-CPR fusion constructs linking SbF6H_trN and ATR2_tr, nine linkers peptides were selected based on the prior literature [16,66,84] and predictions from the VU Centre for Integrative Bioinformatics (IBIVU) linker database (http://www.ibi.vu.nl/programs/linkerdbwww/ (accessed on 6 August 2024)) (Table 2). The selection was based on the linkers’ function, their suitability for particular enzyme classes and their previous applications. These included flexible (LGS), rigid (LPap), and enzyme-specific designs (LMon1, LMon2, and LHyd1), as well as G4S-derived sequences with protease resistance (LL0–LWT).

**Table 2:** Linkers used in this study.

Name	Sequence	Source of Reference
LGS	(GGGS)3	[16,66]
LPap	PAPAP	[16,66]
LMon1	AKQDAY	(http://www.ibi.vu.nl/programs/linkerdbwww/)
LMon2	LEEKVAVLKARAFNEVD	(http://www.ibi.vu.nl/programs/linkerdbwww/)
LHyd1	VPMLADRT	(http://www.ibi.vu.nl/programs/linkerdbwww/)
LL0	A	[84]
LL1	AGARFANGHQH	[84]
LL2	AGARFANGHLALQH	[84]
LWT	AGARFANGHRMLALQHQH	[84]

### 4.5. Clone Selection

Transformants were plated on Lysogeny Broth (LB) agar (1% tryptone, 0.5% yeast extract, 0.5% NaCl, and 2% agar) supplemented with antibiotics (50 µg/mL ampicillin and 30 µg/mL kanamycin or 50 µg/mL gentamicin) and 20 µg/mL X-gal (5-bromo-4-chloro-3-indolyl β-D-galactopyranoside). Colonies were screened via colony PCR using OneTaq DNA Polymerase (New England BioLabs, USA). Plasmids were extracted with a Monarch Plasmid Miniprep Kit (New England BioLabs USA), quantified using a NanoDrop UV5 spectrophotometer (Mettler Toledo, Poland) and verified by Sanger sequencing (Macrogen Europe, Amsterdam, The Netherlands). All constructed plasmids are available upon request.

### 4.6. Media Composition

Pre-cultures were prepared in LB medium supplemented with 50 µg/mL gentamicin and incubated overnight at 37 °C, 120 rpm. Strains were cultivated in 15 mL in three different media, LB, Terrific Broth (TB), and Dynamite Medium (DM) [85], supplemented as required. Detailed composition of all media can be found in the Supplementary Materials.

For activity studies, strains carrying pSbMN-pJ or pSbN-pJ plasmids were grown in 15 mL DM (50 µg/mL gentamicin) to an optical density at 600 nm (OD_600_) of 1.5, supplemented with 0.5 mM 5-aminolevulinic acid (5-ALA), and incubated at 28 °C, 120 rpm. Chrysin (0.1 mM final concentration, from 12 mM DMSO stock) was added 2 h after 5-ALA addition. Reactions were carried out aerobically at 28 °C, 120 rpm for 72 h. 

### 4.7. Supplementation via Bacterial Growth

To assess 5-ALA and rhamnose effects on bacterial growth, *E. coli* strains harboring different CYP constructs (SbN-pJ, GmN-pJ, SbN_LMon2-pJ, SbN_LGS-pJ, SbN_LMon2-prhaB, and SbN_LGS-prhaB) were cultivated in 0.2 mL DM at 28 °C with appropriate antibiotics, 0.5 mM 5-ALA and 15 mM rhamnose (for SbN_LMon2-prhaB and SbN_LGS-prhaB) in a 96-well microplate. Growth was monitored at 600 nm (optical density) every 30 min for 24 h using a Synergy H1 microplate reader (BioTek Instruments, Winooski, VT, USA).

### 4.8. Enzyme Activity Assay

CYP activity was measured indirectly by assessing hydroxylation reaction progress in whole-cell cultures co-expressing CYP and CPR. Constructs included Sb-pJ, SbN-pJ, SbM-pJ, SbMN-pJ, Gm-pJ, GmN-pJ, GmM-pJ, and GmMN-pJ. Aerobic cultures (15 mL DM, 28 °C, 120 rpm) were supplemented with 50 µg/mL gentamicin, 0.5 mM 5-ALA, and 0.1 mM chrysin. Samples of 0.6 mL were taken at 24, 48, and 72 h and extracted with 0.2 mL ethyl acetate, vortexed, and centrifuged (21,300× *g*, 5 min), and 20 µL of the organic phase was mixed with 180 µL of methanol and analyzed by UPLC-DAD.

### 4.9. SDS-Page and Western Blot Analysis

For enzyme expression analysis, the cells were harvested (21,300× *g*, 2 min) and lysed in NEBExpress *E. coli* Lysis Reagent (New England Biolabs, USA), and protein concentration was determined by Bradford assay using BSA as the standard [86]. Then, 10 µg of cell lysates was separated by home-made 10% gel, stained with Coomassie Brilliant Blue (Cepham Life Sciences, Fulton, MD, USA) and analyzed using a GelDoc™ imaging system (BIO-RAD, Hercules, CA, USA). Molecular weights were estimated using Precision Plus Protein™ Unstained Protein Standards (Bio-Rad, USA).

For fusion protein visualization, 0.3 L LB medium supplemented with kanamycin (30 µg/mL) was inoculated with 1% (*v*/*v*) overnight culture and grown at 37 °C with shaking (120 rpm) to OD_600_ = 0.6. Protein expression was induced with 15 mM L-rhamnose (from a 1 M water stock) and 0.5 mM 5-ALA (from a 0.1 M water stock), followed by incubation at 28 °C overnight (120 rpm). Next, cells were harvested by centrifugation (4000× *g*, 15 min), washed, and resuspended in 12 mL of 50 mM Tris–HCl buffer (150 mM NaCl, 10 mM β-mercaptoethanol, pH 8.0). Lysozyme was added (1.5 mL of 10 mg/mL in Tris–HCl buffer), and the suspension was incubated at room temperature for 30 min with gentle mixing. Cells were disrupted by sonication (6 min: 30 s pulses with 30 s pauses). Cell debris was removed by centrifugation (14,000× *g*, 15 min, 4 °C). The resulting pellet was resuspended in 18 mL of denaturing buffer (10 mM Tris–HCl, 100 mM NaH_2_PO_4_, 8 M urea, pH 8.0) and incubated overnight with stirring. After centrifugation (14,000× *g*, 30 min), the supernatant was analyzed by SDS–PAGE using a 7.5% polyacrylamide gel. Molecular weight estimation was performed using Precision Plus Protein™ Unstained Protein Standards (Bio-Rad, USA) for Coomassie Brilliant Blue staining and the PageRuler™ Prestained Protein Ladder (Thermo Fisher Scientific, USA) for Western blot analysis.

### 4.10. P450 Measurement

The level of 450 expression in the P450 mutant library was determined based on whole-cell P450 concentrations in *E. coli* according to the protocol described by Johnston W.A. et al. [87].

### 4.11. Time-Course Analysis of Baicalein Production

To monitor baicalein production and stability over time, cultures harboring SbN-prhaB and SbN-pJ plasmids were grown aerobically in 50 mL DM (50 µg/mL gentamicin, 0.5 mM 5-ALA, and 15 mM rhamnose (SbN-prhaB)) at 28 °C with 0.1 mM baicalein or chrysin, respectively. Samples were collected over 48 h and analyzed as described above.

### 4.12. Flavonoid Stabilization

Chrysin and baicalein concentrations were measured in whole-cell cultures of *E. coli* DH 10-beta with and without co-expression of CYP and CPR (Sb-pJ). Aerobic cultures (30 mL DM, 28 °C, 120 rpm) were supplemented with 50 µg/mL gentamicin and 0.5 mM 5-ALA. After 24 h of cultivation, baicalein and chrysin were added to the cultures together with 25 mM dithiothreitol (DTT). Additionally, flavonoid concentrations were measured in DM alone as a control. Samples of 0.6 mL were collected at 24, 48, and 72 h and analyzed as described above.

To assess the effects of DTT on bacterial growth, *E. coli* strains harboring the SbN-pJ CYP construct and control strains lacking CYP were cultivated in 0.2 mL DM at 28 °C with 50 µg/mL gentamicin and 0.5 mM 5-ALA in a 96-well microplate. Growth was monitored at 600 nm (optical density) every 30 min for 24 h using a Synergy H1 microplate reader (BioTek Instruments, USA).

### 4.13. Resting-Cell Reaction

SbN-prhaB and SbN-LGS-prhaB strains were grown in 100 mL LB medium (1% inoculum) supplemented with kanamycin (30 µg/mL) at 37 °C to OD_600_ = 0.6. Protein expression was induced with 15 mM L-rhamnose (from a 1 M water stock) and 0.5 mM 5-aminolevulinic acid (5-ALA) (from a 0.1 M water stock), followed by incubation at 28 °C overnight with shaking. Next, cells were harvested by centrifugation, washed, and resuspended in 25 mM phosphate buffer (pH 7.8) to the original OD_600_ value. Reactions were performed in 15 mL of resting-cell suspension at 28 °C and 120 rpm. Chrysin was added from a 12 mM DMSO stock solution to a final concentration of 0.1 mM. After 24 h, the flasks of each strain were divided into two groups and dithiothreitol (DTT) was added to one of them to a final concentration of 25 mM. Samples were collected after 2, 4, 6, 21, 24, and 48 h and analyzed as described previously.

### 4.14. Chemical Analysis

UPLC-DAD analysis was performed on an Ultimate 3000 system (Dionex, Sunnyvale, CA, USA) equipped with a diode array detector and controlled by Chromeleon 6.80 software following the previously described procedure [83]. Separation was carried out on an Acclaim RSLC Polar Advantage II column (2.1 mm × 100 mm, 2.2 μm) with a pre-column, using a linear gradient of 0.01% formic acid in water (A) and acetonitrile (B): 0 min, 15% B; 0–4.2 min, 98% B; 4.2–6.0 min, 15% B. The flow rate was 0.7 mL/min, the injection volume was 10 μL, and the column temperature was 28 °C. Detection was performed at 330 nm, with UV–Vis spectra recorded in the range of 200–600 nm. Compounds were identified by retention time and UV spectra compared with authentic standards.

LC-MS analysis was carried out on an LC-MS 8045 system (Shimadzu, Kyoto, Japan) with a triple quadrupole and diode array detector, operated by LabSolutions software version 5.120. Separation was performed on a Kinetex C18 column (3 mm × 100 mm, 2.6 µm, 100 Å) with a pre-column, using water with 0.01% formic acid (A) and acetonitrile (B) as mobile phases (0′—15% B; 0 -> 3′—15% B; 3′ -> 10′—100% B; 10′ -> 17.5′—100% B; 17.5′ -> 20′—15% B; 20′-21′—15% B). The flow rate was 0.4 mL/min, the injection volume was 2 µL, and the column temperature was 30 °C. MS operating parameters were: nebulizing gas 3 L/min, heating gas 10 L/min, drying gas 10 L/min, and interface temperature 300 °C. Samples were analyzed in negative ionization using product ion scan analysis with Q1 molecular masses of samples and products [−H^+^]. Collision energy was 35V and the Q3 acquisition range *m*/*z* 80 analyzed compound mass [−H^+^]. Compounds were identified based on molecular mass and fragmentation.

### 4.15. Statistical Analysis

All measurements were performed in triplicate, unless otherwise specified. Data are summarized as mean and standard deviation and analyzed for statistical significance with Statistica software (version 13.3) by unpaired *t*-test and one-way analysis of variance (one-way ANOVA) with Tukey’s post hoc test. Equality of the variance was verified using Levene’s test. If needed, a Box–Cox transformation was performed to unify the variance of the data.

## 5. Conclusions

In conclusion, this work underscores the intricate interplay between genetic engineering, host strain selection, and bioprocess conditions in harnessing plant P450 enzymes for efficient biosynthesis of valuable flavonoids such as baicalein. In future studies integrating advanced linker design, host cell engineering focused on deletion of possible degrading enzymes, immobilization strategies, and the exploration of alternative redox partners will be instrumental in stabilizing both the enzyme and product, thereby unlocking the full biotechnological potential of recombinant P450 monooxygenases. Our results also demonstrate that degradation processes affecting substrates and products constitute a critical bottleneck in microbial flavonoid biosynthesis or biotransformation and should therefore be systematically addressed at the earliest stages of experimental design, as they may hinder not only the final yield but also result in higher variance between the samples tested.

## Data Availability

The original contributions presented in this study are included in the article/supplementary material. Further inquiries can be directed to the corresponding author(s).

## Supplementary Materials

The following supporting information can be downloaded at https://www.mdpi.com/article/10.3390/molecules31122189/s1, Figure S1: Protein sequence analysis of (a) SbF6H and (b) GmF6H; Figure S2: Effect of 5-aminolevulinic acid (5-ALA) on the growth of bacterial strains; Figure S3: In vivo baicalein formation by SbF6H_trN-ATR2_tr fusion proteins; Figure S4: Western blot analysis of SbF6H_trN-ATR2_tr fusion proteins; Figure S5: (a) Degradation of baicalein during 24 h bacterial cultivation, (b) product–substrate correlation during 24 h in vivo reaction; Figure S6: Effect of dithiotreitol on the growth of *E. coli* DH 10-beta strain; Figure S7: LC-MS/MS chromatogram and product fragmentation spectra of reaction SbN-pJ with chrysin; Figure S8: LC-MS/MS chromatogram and product fragmentation spectra of reaction SbN-pJ with apigenin; Figure S9: LC-MS/MS chromatogram and product fragmentation spectra of reaction SbN-pJ with naringenin; Figure S10: LC-MS/MS chromatogram and product fragmentation spectra of reaction SbN-pJ with kaempferol; Figure S11: LC-MS/MS chromatogram and product fragmentation spectra of reaction SbN-pJ with luteolin; Figure S12: LC-MS/MS chromatogram and product fragmentation spectra of reaction SbN-pJ with isoxanthohumol; Table S1: Oligonucleotides, template DNA, vectors, and bacterial strains; Table S2: Plasmid maps of construct used in this study; Table S3: Structure of substrate used in this work; Table S4: Results of one-way ANOVA with Levene’s test for homogeneity of variances and Tukey’s HSD post hoc analysis of cross-reactivity test assessing bacterial strain growth with and without supplementation after 18 h of cultivation; Table S5: Results of one-way ANOVA with Levene’s test for homogeneity of variances and Tukey’s HSD post hoc analysis of cross-reactivity test assessing baicalein production by N-terminal variants of SbF6H and GmF6H after 24 h of in vivo reaction; Table S6: Results of one-way ANOVA with Levene’s test for homogeneity of variances and Tukey’s HSD post hoc analysis of cross-reactivity test assessing baicalein production by two modified SbF6H variants in different *E. coli* host strains after 24 h of in vivo reaction; Table S7: Results of one-way ANOVA with Levene’s test for homogeneity of variances and Tukey’s HSD post hoc analysis of cross-reactivity test assessing baicalein production by SbF6H_trN-ATR2_tr fusion proteins linked by different linkers after 24 h of in vivo reaction; codon-optimized sequences, media composition.

## Abbreviations

SbF6H	hydroxylase from *Scutellaria baicalensis*
GmF6H	hydroxylase from *Glycime max*
5-ALA	5-aminolevulinic acid
Rhm	rhamnose
