# Supplementary material for: P450 Fusion Protein Expressed in E. coli for Regioselective Hydroxylation of Flavonoids
Source: Molecules. 2026 Jun 22;31(12):2189. doi: 10.3390/molecules31122189 (PMC13304987; doi:10.3390/molecules31122189)
Supplement: Supplementary file 1 [file molecules-31-02189-s001.zip › molecules-4286958-supplementary.pdf]

P450 fusion protein expressed in *E. coli* for regioselective hydroxylation of flavonoids

Kinga Dulak¹, Agata Matera¹, Sandra Sordon¹, Maciej Wolak¹, Kinga Hyla², Ewa Huszcza¹, Jarosław Popłoński^{1*}

- 1 Department of Food Chemistry and Biocatalysis, Wrocław University of Environmental and Life Sciences, Wrocław, Poland; kinga.dulak@upwr.edu.pl, agata.matera@upwr.edu.pl, sandra.sordon@upwr.edu.pl, maciekwolak96@gmail.com, ewa.huszcza@upwr.edu.pl, jaroslaw.poplonski@upwr.edu.pl
- 2 Department of Biotechnology and Food Microbiology, Wrocław University of Environmental and Life Sciences, Wrocław, Poland: kinga.hyla@upwr.edu.pl
- * jaroslaw.poplonski@upwr.edu.pl

List of content

**Supplementary Figure S1.** Protein sequence analysis of (a) SbF6H, and (b) GmF6H.....	2
**Supplementary Figure S2.** Effect of 5-aminolevulinic acid (5-ALA) on the growth of bacterial strains.....	3
**Supplementary Figure S3.** <i>In vivo</i> baicalein formation by SbF6H_trN-ATR2_tr fusion proteins.....	3
**Supplementary Figure S4.** Western blot analysis of SbF6H_trN-ATR2_tr fusion proteins.....	4
**Supplementary Figure S5.** (a) Degradation of baicalein during 24-hour bacterial cultivation, (b) Product-substrate correlation during a 24-hour <i>in vivo</i> reaction.....	4
**Supplementary Figure S6.** Effect of dithiotreitol on the growth of <i>E. coli</i> DH 10-beta strain.....	5
**Supplementary Figure S7.** LC-MS/MS chromatogram and product fragmentation spectra of reaction SbN-pJ with chrysin	5
**Supplementary Figure S8.** LC-MS/MS chromatogram and product fragmentation spectra of reaction SbN-pJ with apigenin.....	6
**Supplementary Figure S9.** LC-MS/MS chromatogram and product fragmentation spectra of reaction SbN-pJ with naringenin.....	6
**Supplementary Figure S10.** LC-MS/MS chromatogram and product fragmentation spectra of reaction SbN-pJ with kaempferol.....	7
**Supplementary Figure S11.** LC-MS/MS chromatogram and product fragmentation spectra of reaction SbN-pJ with luteolin	7
**Supplementary Figure S12.** LC-MS/MS chromatogram and product fragmentation spectra of reaction SbN-pJ with isoxanthohumol.....	8
**Supplementary Table S1.** Oligonucleotides, template DNA, vectors, and bacterial strains	8
**Supplementary Table S2.** Plasmid maps of construct used in this study.....	13
**Supplementary Table S3.** Structure of substrates used in this work.....	22
**Supplementary Table S4.** Results of one-way ANOVA with Levene's test for homogeneity of variances and Tukey's HSD post hoc analysis of cross-reactivity test assessing bacterial strains growth with and without supplementation after 18 h of cultivation.....	25

**Supplementary Table S5.** Results of one-way ANOVA with Levene's test for homogeneity of variances and Tukey's HSD post hoc analysis of cross-reactivity test assessing baicalein production by N-terminal variants of SbF6H and GmF6H after 24 h of <i>in vivo</i> reaction.....	25
**Supplementary Table S6.** Results of one-way ANOVA with Levene's test for homogeneity of variances and Tukey's HSD post hoc analysis of cross-reactivity test assessing baicalein production by two modified SbF6H variants in different <i>E. coli</i> host strains after 24 h of <i>in vivo</i> reaction.....	26
**Supplementary Table S7.** Results of one-way ANOVA with Levene's test for homogeneity of variances and Tukey's HSD post hoc analysis of cross-reactivity test assessing baicalein production by SbF6H_trN-ATR2_tr fusion proteins linked by different linkers after 24 h of <i>in vivo</i> reaction.....	26
Codon optimized sequences.....	27
Media composition.....	28

a

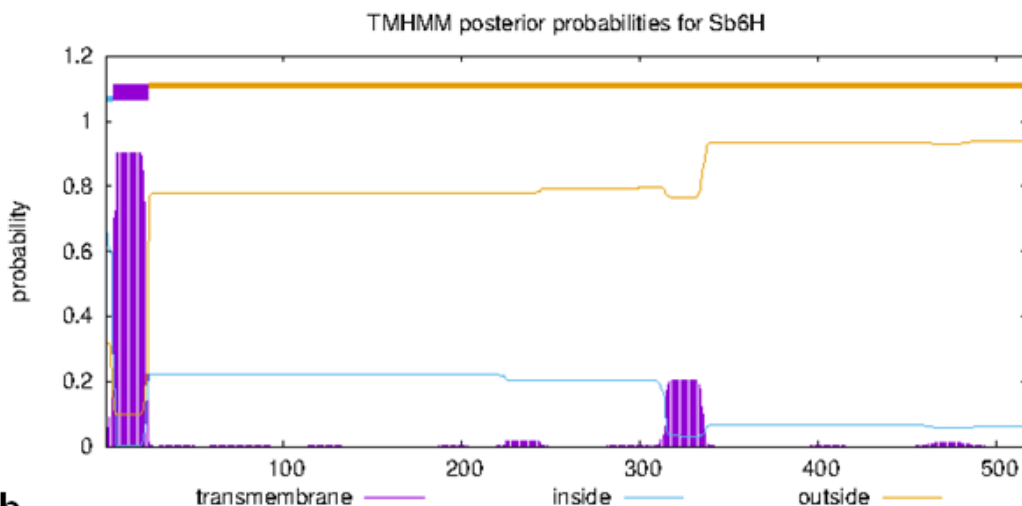

b

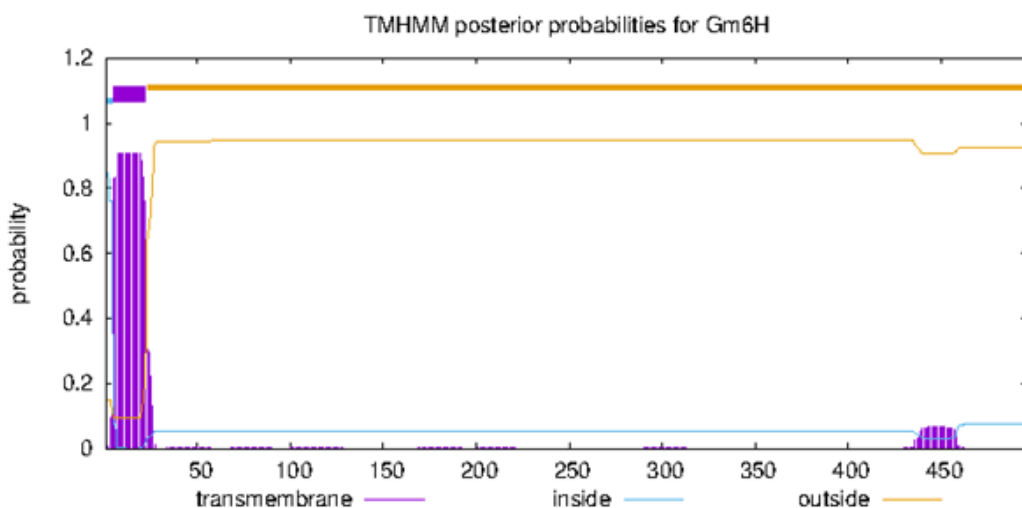

Supplementary Figure S1. Protein sequence analysis of (a) Sb6H, and (b) Gm6H visualized using TMHMM software.

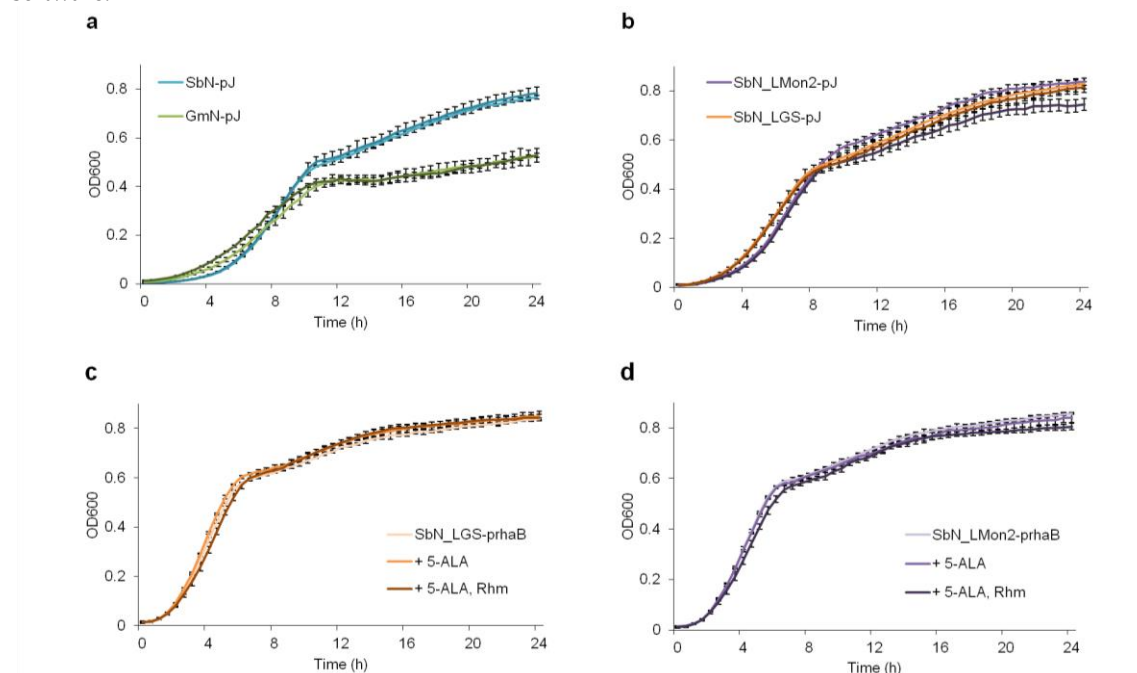

Supplementary Figure S2. Effect of 5-aminolevulinic acid (5-ALA) on the growth of bacterial DH 10-beta strains harboring (a) pSbN-pJ and pGmN-pJ plasmid under constitutive promoters, (b) pSbN_LMon2-pJ and pSbN_LGS-pJ plasmid under constitutive promoters, (c) pSbN-LGS-prhaB and (d) pSbN_LMon2-prhaB plasmid under a rhamnose-inducible promoter. In panels (a) and (b), light-colored curves represent cultures supplemented with 0.5 mM 5-ALA, while dark-colored curves represent cultures grown without 5-ALA.

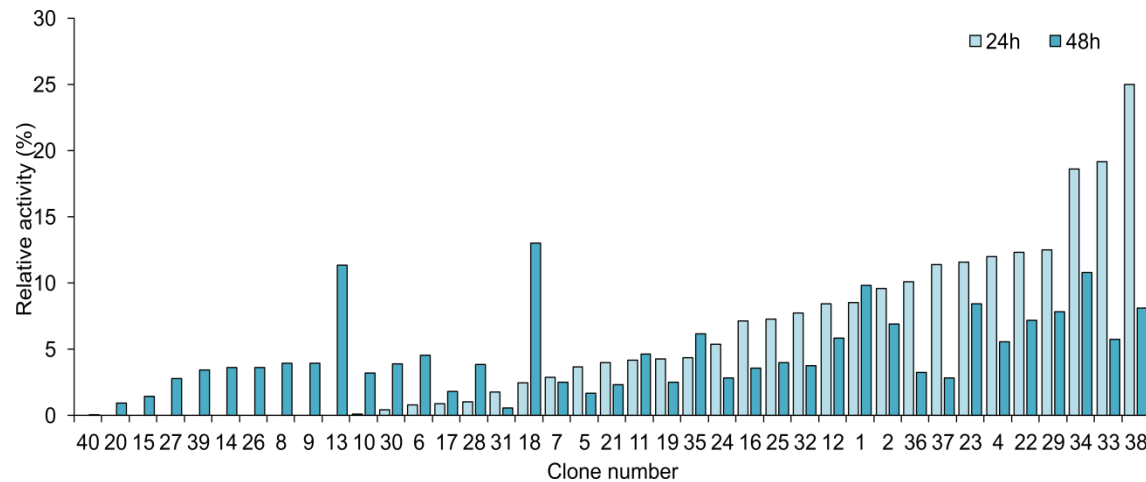

Supplementary Figure S3. *In vivo* baicalein formation after 24 and 48 hours by SbF6H_trN-ATR2_tr fusion proteins linked by different linkers. No product was detected for clone nr 3. Chrysin was used as a substrate at an initial concentration of 0.1 mM.

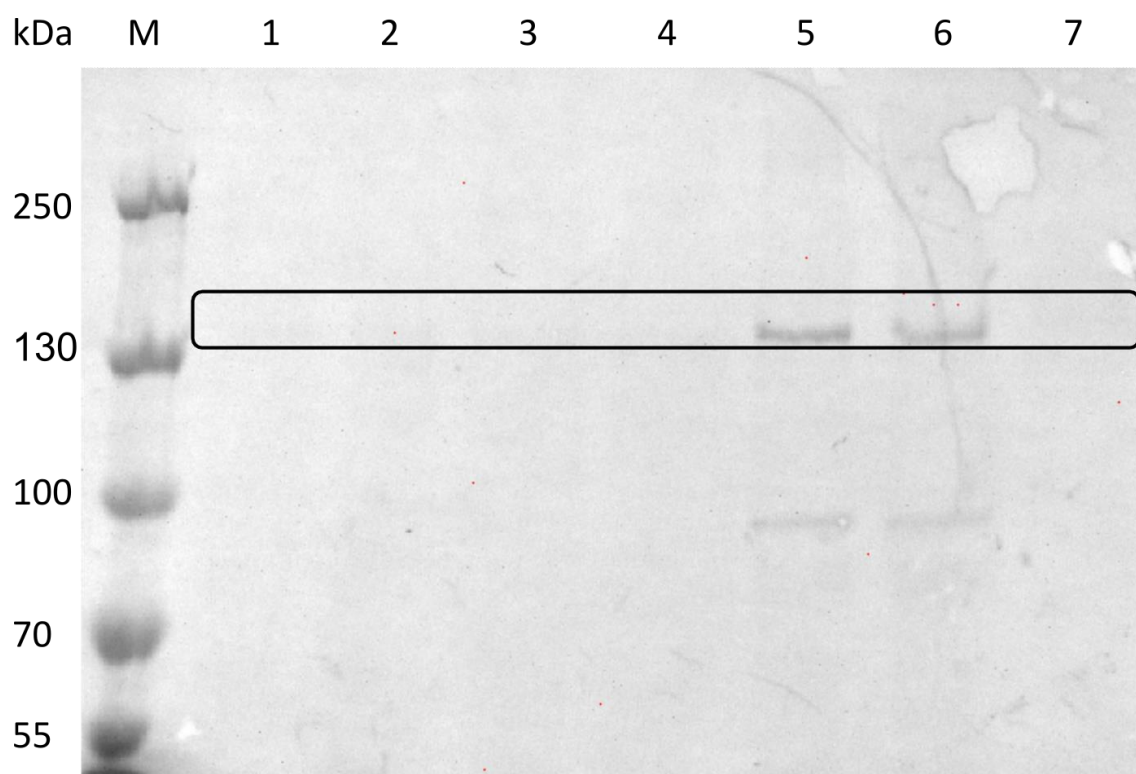

Supplementary Figure S4. Western blot analysis of crude extracts containing CYP-CPR enzyme pairs co-expressed as fusion protein linked by different linkers. The frames highlight the enzyme fraction CYP-CPR fusion protein, ladder (M), SbN_LGS-prhaB (line 1), SbN_LMon1-prhaB (line 2), SbN_LMon2-prhaB (line 3), SbN_LHyd1-prhaB (line 4), SbN_LL1-prhaB (line 5), SbN_LL2-prhaB (line 6), SbN_LWT-prhaB (line 7). Expected mass of recombinant protein: Sb6H_trN and ATR_tr genes fusion with linker: L_GS – 128.5 kDa, L_Mon1 – 128.4 kDa, L_Mon2 – 129.5 kDa, L_Hyd1 – 128.5 kDa, L_L1 – 128.5 kDa, L_L2 – 129.1 kDa, L_WT – 129.6 kDa.

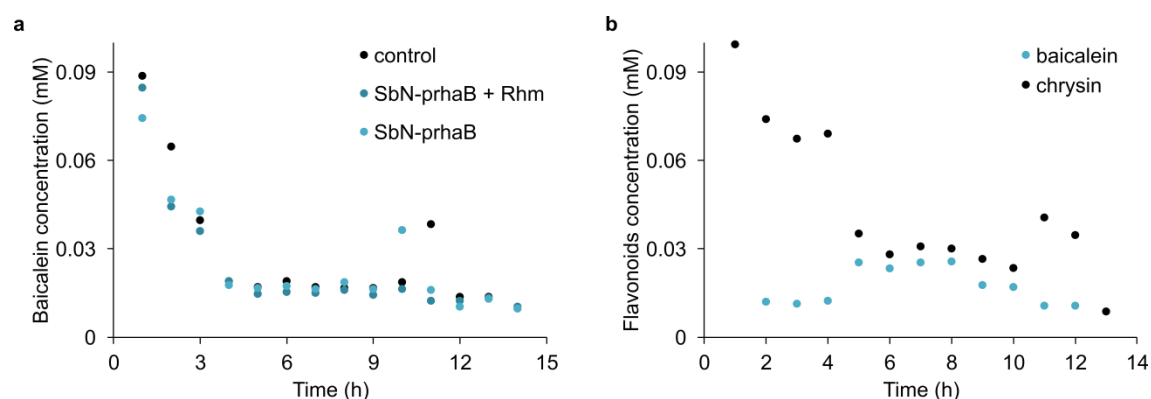

Supplementary Figure S5. (a) Degradation of baicalein during 48-hour bacterial cultivation with and without CYP-CPR expression. Baicalein was used as a substrate at an initial concentration of 0.1 mM. (b) Product-substrate ratio during a 24-hour *in vivo* reaction. Chrysin was used as a substrate at an initial concentration of 0.1 mM.

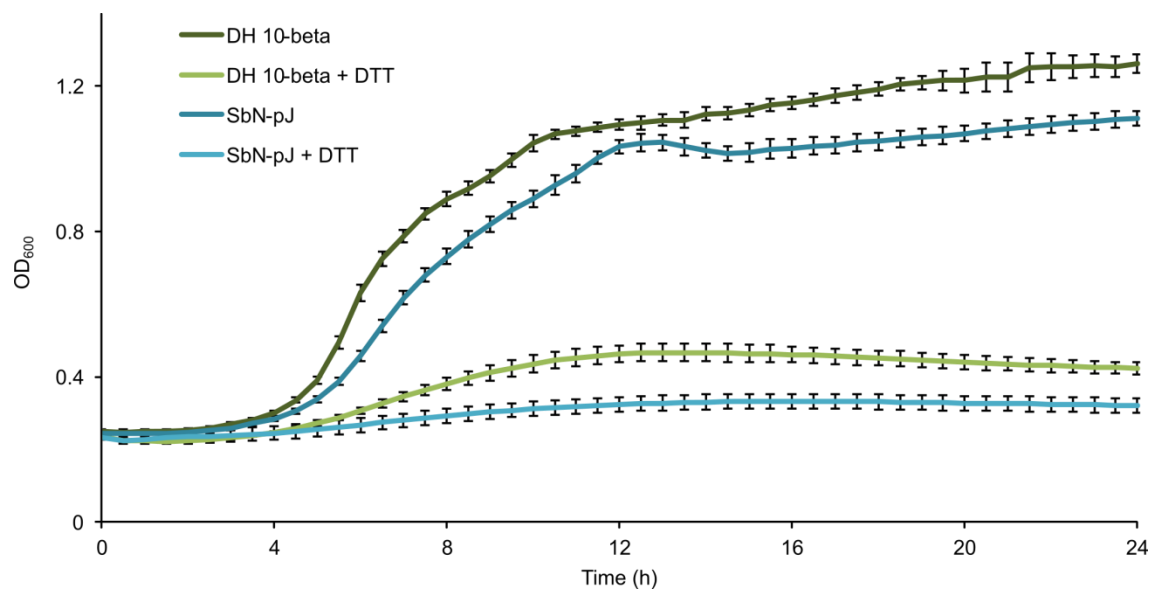

Supplementary Figure S6. Effect of dithiothreitol (DTT) on the growth of *E. coli* DH 10-beta strains harboring or lacking the pSbN-pJ plasmid under constitutive promoters.

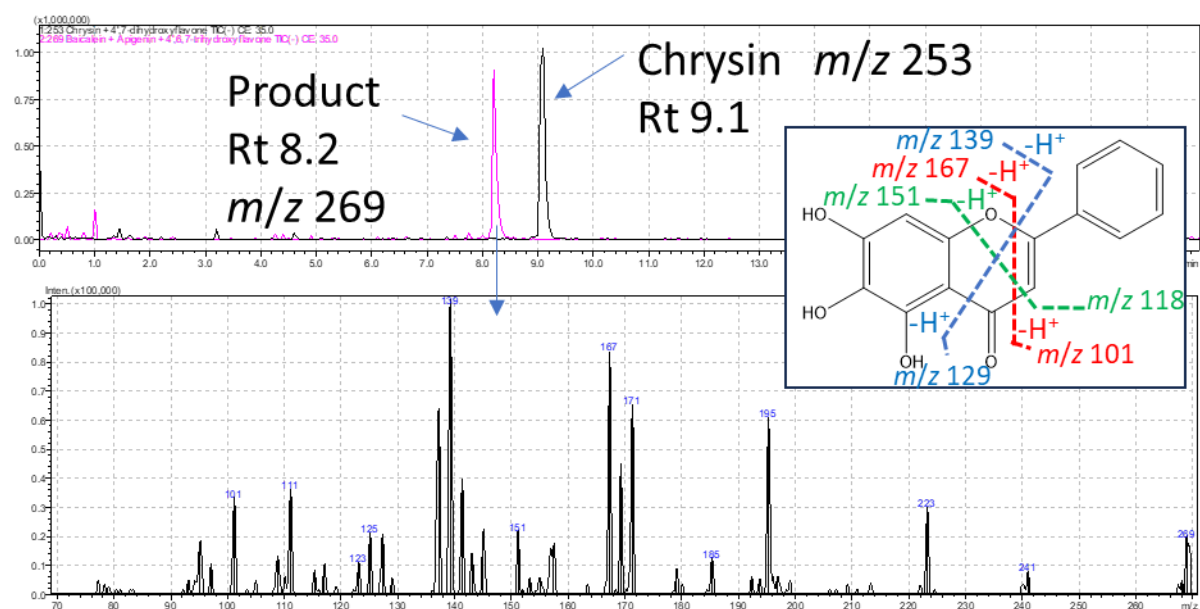

Supplementary Figure S7. LC-MS/MS chromatogram and product fragmentation spectra of reaction SbN-pJ with chrysin.

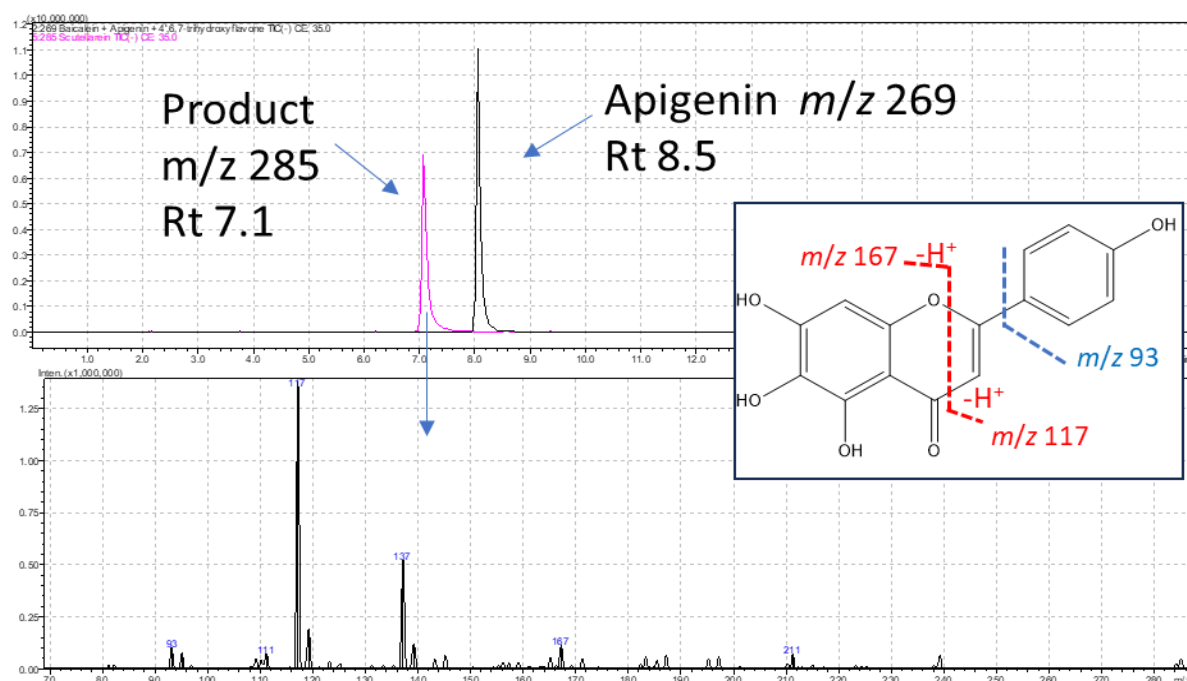

Supplementary Figure S8. LC-MS/MS chromatogram and product fragmentation spectra of reaction SbN-pJ with apigenin.

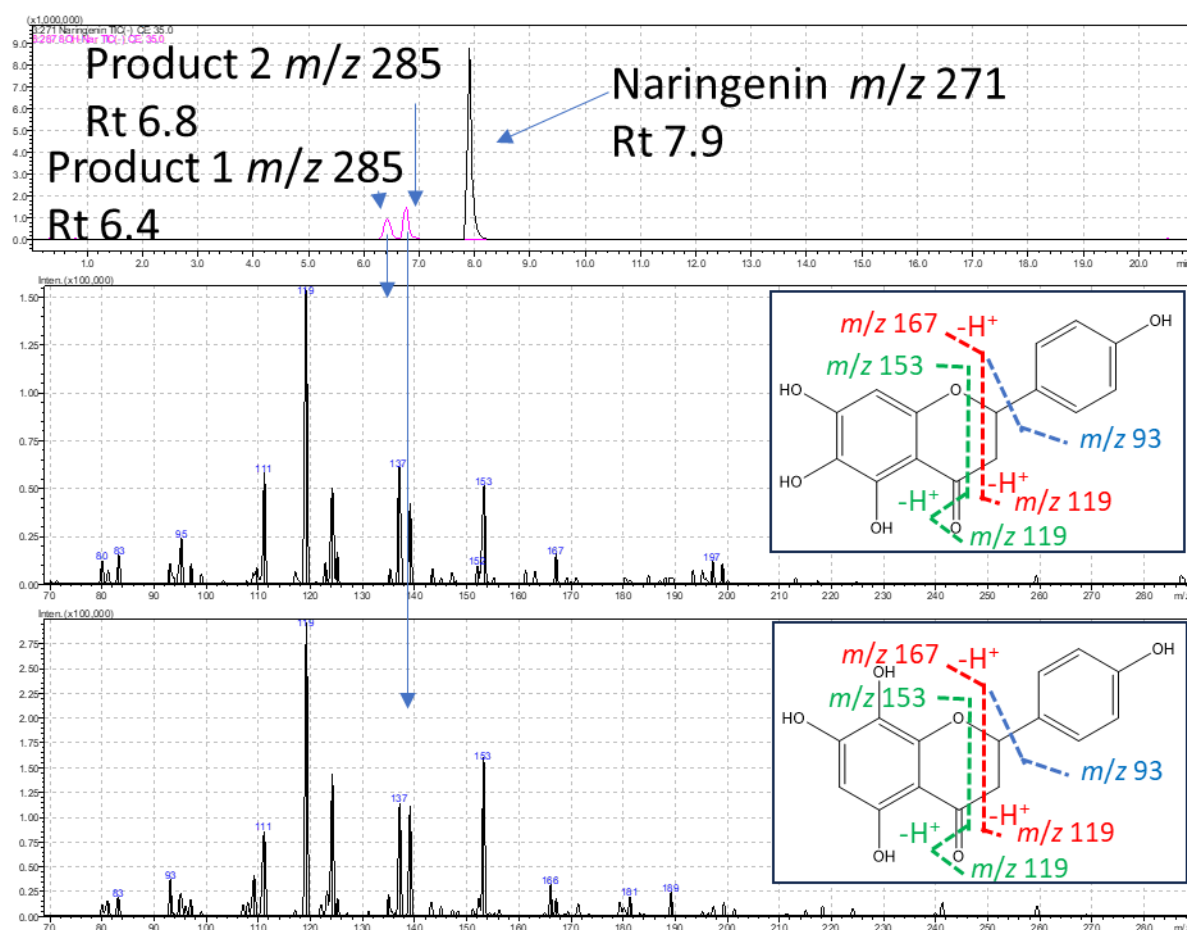

Supplementary Figure S9. LC-MS/MS chromatogram and product fragmentation spectra of reaction SbN-pJ with naringenin.

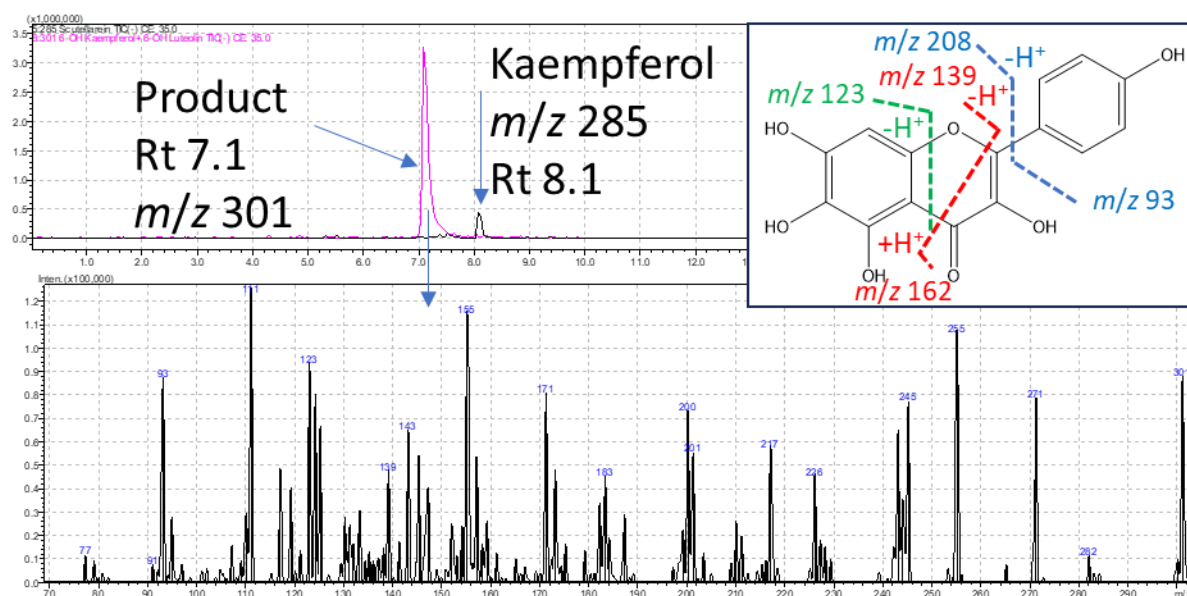

Supplementary Figure S10. LC-MS/MS chromatogram and product fragmentation spectra of reaction SbN-pJ with kaempferol.

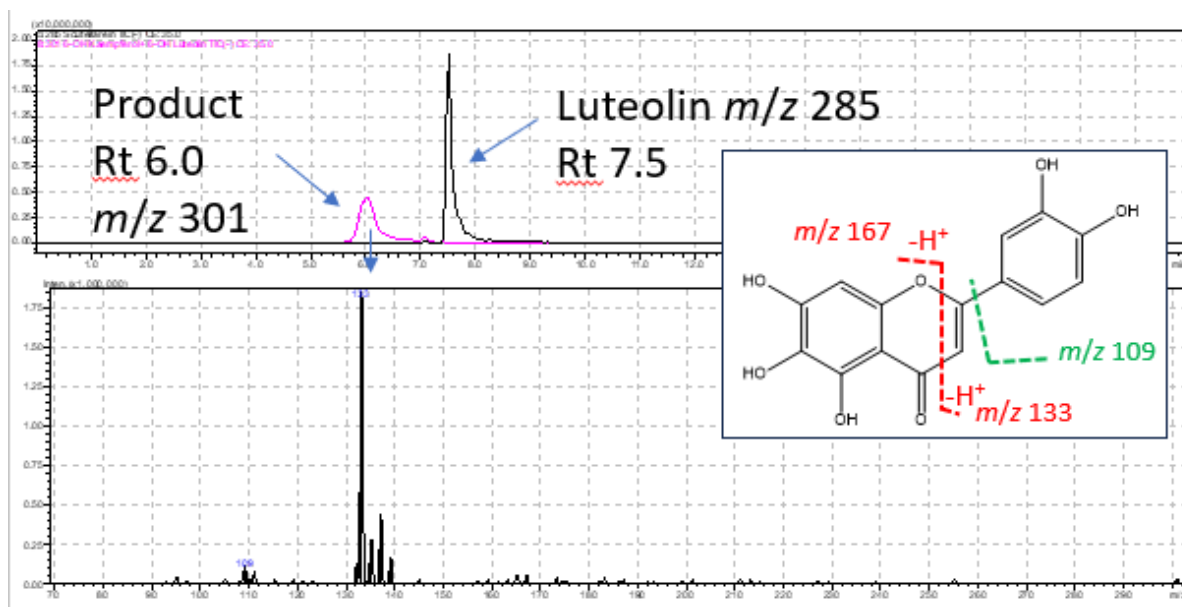

Supplementary Figure S11. LC-MS/MS chromatogram and product fragmentation spectra of reaction SbN-pJ with luteolin.

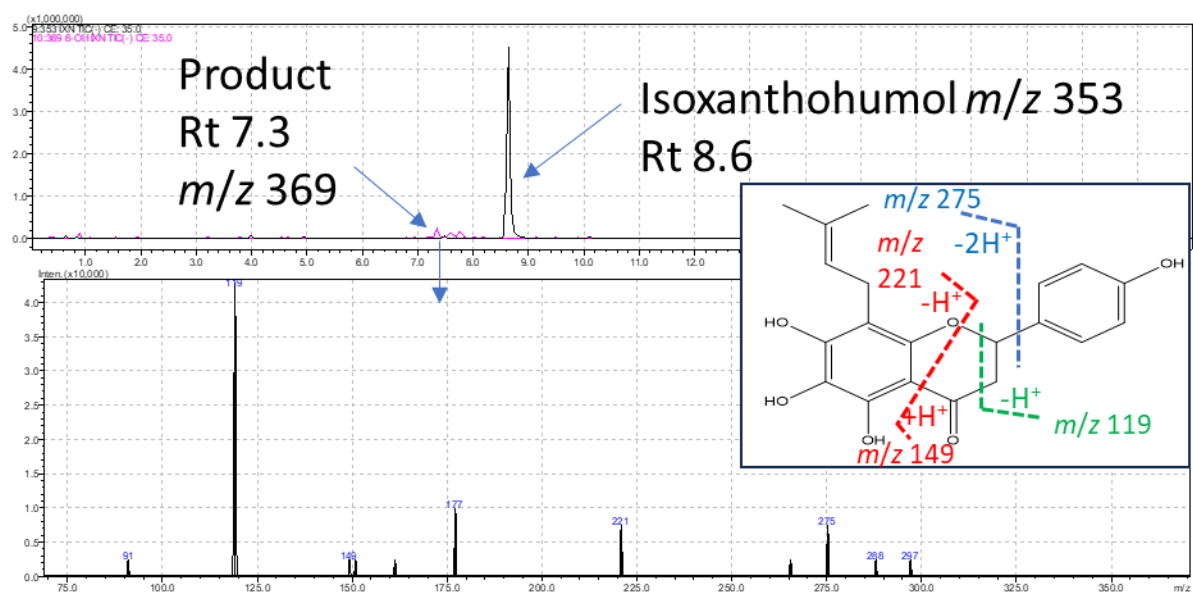

Supplementary Figure S12. LC-MS/MS chromatogram and product fragmentation spectra of reaction SbN-pJ with isoxanthohumol. Please note, that hydroxylation site in this molecule may be different than depicted.

Supplementary Table S1. Oligonucleotides, template DNA, vectors, and bacterial strains used in this study.

Name	Properties	Source or reference
<i>Genes</i>		
<i>Sb6H</i>	hydroxylase from <i>Scutellaria baicalensis</i>	ASW21050
<i>Gm6H</i>	hydroxylase from <i>Glycine max</i>	NP_001304582.1
<i>ATR2</i>	reductase from <i>Arabidopsis thaliana</i>	CAA46815
<i>Oligonucleotides</i>		
R24_SEVA182	AGCGGATAACAATTTACACAGGA	[1]
F24_SEVA182	CGCCAGGGTTTTCCAGTCACGAC	[1]
Sb6H_Ntr_Fwd	GGTCTCAAATGAAACCGAAAAAAGCAGCC<br>TGAACGC	This study
Sb6H_Rev	GGTCTCTAAGCTTAATACAGGGTCGGGCTC	This study
Gm6H_Ntr_Fwd	GGTCTCAAATGAAAATTGTGACCAAAAAA<br>GCAACAGCAC	This study
Gm6H_Rev	GGTCTCTAAGCTTAGTTATGCACGGTTTTTC	This study

N-MALLLAVF_Fwd	GGTCTCACCATATGGCACTGCTGCTGGCGGT<br>GTTTGCAATGAGAGACC	This study
N-MALLLAVF_Rev	GGTCTCTCATTGCAAACACCGCCAGCAGCA<br>GTGCCATATGGTGAGACC	This study
AtRtr_F1	GCGAAACCGTTGATGAAGCACTGC	This study
AtRtr_F2	GCATGGATAGCACCAAAGCCG	This study
AtRtr_R1	GCTATATTCATCAACTTTACC	This study
AtRtr_R2	GCGGTGCCGGTCTGGGTGCC	This study
Sb6H_Fwd_D	GGTCTCAAATGGAAGTGAAGCAGCG	This study
Sb6H_Rv_E	GGTCTCACCTATACAGGGTCGGGCTCAGGC	This study
Gm6H_Fv_D	GGTCTCAAATGGATCTGCAGCTGC	This study
Gm6H_Rv_E	GGTCTCACCTATTATGCACGGTTTTTCGG	This study
Atr_Fw_F	GGTCTCATTCGGTATGGGTAGCGGTAATAGC<br>AAACG	This study
Atr_Rv_G	GGTCTCTAAGCTTACCAAACATCACGCAGAT<br>AACG	This study
L_GS_Fw	GGTCTCATAGGGGCGGGGGAGGCAGCGGGG<br>GAGGTGGGAGCGGTGGGGTGGCAGCTTCG<br>AGAGACC	This study
L_GS_Rv	GGTCTCTCGAAGCTGCCACCCCCACCGCTCC<br>CACCTCCCCCGCTGCCTCCCCGCCCCTATG<br>AGACC	This study
L_Mon1_Fw	GGTCTCATAGGGCGAAACAGGATGCTTATTG<br>GTTCGAGAGACC	This study
L_Mon1_Rv	GGTCTCTCGAACCAATAAGCATCCTGTTTCG<br>CCCTATGAGACC	This study
L_Mon2_Fw	GGTCTCATAGGCTCGAGGAAAAGGTGGCGG<br>TACTCAAAGCCCGTGCTTTTAACGAGGTAGA<br>TTTCGAGAGACC	This study
L_Mon2_Rv	GGTCTCTCGAAATCTACCTCGTTAAAAGCAC<br>GGGCTTTGAGTACCGCCACCTTTTCCTCGAG<br>CCTATGAGACC	This study
L_Hyd1_Fw	GGTCTCATAGGGTGCCTATGCTGGCGGATCG<br>CACCTTCGAGAGACC	This study

L_Hyd1_Rv	GGTCTCTCGAAGGTGCGATCCGCCAGCATA<br>GGCACCTATGAGACC	This study
L_Pap_Fw	GGTCTCATAGGCCGGCACCAGCGCCGTTTCG<br>AGAGACC	This study
L_Pap_Rv	GGTCTCTCGAACGGCGCTGGTGCCGGCCTAT<br>GAGACC	This study
L_L0_Fw	GGTCTCATAGGGGCTTCGAGAGACC	This study
L_L0_Rv	GGTCTCTCGAAGCCCCTATGAGACC	This study
L_L1_Fw	GGTCTCATAGGGTGCTGGTGACCATTGGCGA<br>ACCGAGCACCCGCTTCGAGAGACC	This study
L_L1_Rv	GGTCTCTCGAAGCGGGTGCTCGGTTCGCCAA<br>TGGTCACCAGCACCTATGAGACC	This study
L_L2_Fw	GGTCTCATAGGGTGCTGCAGCGCCAGGTGA<br>CCATTGGCGAACCGAGCACCCGCTTCGAGA<br>GACC	This study
L_L2_Rv	GGTCTCTCGAAGCGGGTGCTCGGTTCGCCAA<br>TGGTCACCTGGCGCTGCAGCACCTATGAGA<br>CC	This study
L_WT_Fw	GGTCTCATAGGGTGCTGGTGCTGCAGCGCCA<br>GCATCCGGTGACCATTGGCGAACCGAGCAC<br>CCGCTTCGAGAGACC	This study
L_WT_Rv	GGTCTCTCGAAGCGGGTGCTCGGTTCGCCAA<br>TGGTCACCGGATGCTGGCGCTGCAGCACCA<br>GCACCCTATGAGACC	This study
<i>Vector</i>		
pSEVA88c1	pUC-pIJ101 ori, AmpR	[2]
pSEVA23g19g1	pBBR1 ori, KmR	[3]
pSEVA23g19g2	pBBR1 ori, KmR	[3]
pSEVA63g19gA	pBBR1 ori, GmR	[3]
pRhaBAD_12 vector	pSEVA23g19g1 carrying RhaBAD_cassette	[4]
<i>Bacterial strain</i>		
DH5-alpha	F' proA+B + lacIq Δ(lacZ)M15 zzf::Tn10 (TetR) /<br>fhuA2Δ(argF-lacZ)U169 phoA glnV44<br>Φ80Δ(lacZ)M15 gyrA96 recA1 relA1 endA1 thi-1<br>hsdR17	New England<br>Biolabs Inc.

DH10-beta	Δ (ara-leu) 7697 araD139 fhuA Δ lacX74 galK16 galE15 e14- ϕ 80dlacZ Δ M15 recA1 relA1 endA1 nupG rpsL (StrR) rph spoT1 Δ (mrr-hsdRMS-mcrBC)	New England Biolabs Inc.
BL21 (DE3)	fhuA2 [lon] ompT gal (λ DE3) [dcm] Δ hsdS λ DE3 = λ sBamHI Δ EcoRI-B int::(lacI::PlacUV5::T7 gene1) i21 Δ nin5	New England Biolabs Inc.
Rosetta (DE3)	F' ompT hsdSB(rB- mB-) gal dcm (DE3) pLysSRARE2 (CamR)	Novagen, Merck Millipore
Arctic Express (DE3)	F' ompT hsdS(rB' mB') dcm+ Tetr gal λ (DE3) endA Hte [cpn10 cpn60 GentR]	Agilent Technologies
<i>Transcriptional units</i>		
Sb6H	KmR, pBBR1, Lv1 pSEVA23g19g1 vector, (pJ23100, RBS T7 from pET28, **Sb6H** , T7 terminator from pET28)	This study
Sb6H_trN	KmR, pBBR1, Lv1 pSEVA23g19g1 vector, (pJ23100, RBS T7 from pET28, **Sb6H_trN** , T7 terminator from pET28)	This study
N-MAL, Sb6H	KmR, pBBR1, Lv1 pSEVA32g19g1 vector, (pJ23100, RBS T7 from pET28, **N-MALLAVF** , **Sb6H** , T7 terminator from pET28)	This study
N-MAL, Sb6H_trN	KmR, pBBR1, Lv1 pSEVA23g19g1 vector, (pJ23100, RBS T7 from pET28, **N-MALLAVF** , **Sb6H_trN** , T7 terminator from pET28)	This study
Gm6H	KmR, pBBR1, Lv1 pSEVA23g19g1 vector, (pJ23100, RBS T7 from pET28, **Gm6H** , T7 terminator from pET28)	This study
Gm6H_trN	KmR, pBBR1, Lv1 pSEVA23g19g1 vector, (pJ23100, RBS T7 from pET28, **Gm6H_trN** , T7 terminator from pET28)	This study
N-MAL, Gm6H	KmR, pBBR1, Lv1 pSEVA23g19g1 vector, (pJ23100, RBS T7 from pET28, **N-MALLAVF** , **Gm6H** , T7 terminator from pET28)	This study
N-MAL, Gm6H_trN	KmR, pBBR1, Lv1 pSEVA23g19g1 vector, (pJ23100, RBS T7 from pET28, **N-MALLAVF** , **Gm6H_trN** , T7 terminator from pET28)	This study
ATR_tr	KmR, pBBR1, Lv1 pSEVA23g19g1 vector, (pJ23102, RBS T7 from pET28, **ATR_tr** , T7 terminator from pET28)	This study

Sb-pJ	GmR, pBBR1, Lv2 pSEVA63g19gA vector, (pJ23100, RBS T7 from pET28, **<i>Sb6H</i>** , T7 terminator from pET28), (pJ23102, RBS T7 from pET28, **<i>ATR_tr</i>** , T7 terminator from pET28)	This study
SbN-pJ	GmR, pBBR1, Lv2 pSEVA63g19gA vector, (pJ23100, RBS T7 from pET28, **<i>Sb6H_trN</i>** , T7 terminator from pET28), (pJ23102, RBS T7 from pET28, **<i>ATR_tr</i>** , T7 terminator from pET28)	This study
SbM-pJ	GmR, pBBR1, Lv2 pSEVA63g19gA vector, (pJ23100, RBS T7 from pET28, **<i>N-MALLAVE</i>** , **<i>Sb6H</i>** , T7 terminator from pET28), (pJ23102, RBS T7 from pET28, **<i>ATR_tr</i>** , T7 terminator from pET28)	This study
SbMN-pJ	GmR, pBBR1, Lv2 pSEVA63g19gA vector, (pJ23100, RBS T7 from pET28, **<i>N-MALLAVE</i>** , **<i>Sb6H_trN</i>** , T7 terminator from pET28), (pJ23102, RBS T7 from pET28, **<i>ATR_tr</i>** , T7 terminator from pET28)	This study
Gm-pJ	GmR, pBBR1, Lv2 pSEVA63g19gA vector, (pJ23100, RBS T7 from pET28, **<i>Gm6H</i>** , T7 terminator from pET28), (pJ23102, RBS T7 from pET28, **<i>ATR_tr</i>** , T7 terminator from pET28)	This study
GmN-pJ	GmR, pBBR1, Lv2 pSEVA63g19gA vector, (pJ23100, RBS T7 from pET28, **<i>Gm6H_trN</i>** , T7 terminator from pET28), (pJ23102, RBS T7 from pET28, **<i>ATR_tr</i>** , T7 terminator from pET28)	This study
GmM-pJ	GmR, pBBR1, Lv2 pSEVA63g19gA vector, (pJ23100, RBS T7 from pET28, **<i>N-MALLAVE</i>** , **<i>Gm6H</i>** , T7 terminator from pET28), (pJ23102, RBS T7 from pET28, **<i>ATR_tr</i>** , T7 terminator from pET28)	This study
GmMN-pJ	GmR, pBBR1, Lv2 pSEVA63g19gA vector, (pJ23100, RBS T7 from pET28, **<i>N-MALLAVE</i>** , **<i>Gm6H_trN</i>** , T7 terminator from pET28), (pJ23102, RBS T7 from pET28, **<i>ATR_tr</i>** , T7 terminator from pET28)	This study
SbN-prhaBAD	GmR, pBBR1, Lv2 pSEVA63g19gA vector, containing RhaS gene (rhaBAD promoter, RBS T7 from pET28, **<i>Sb6H_trN</i>** , T7 terminator from pET28), (rhaBAD promoter, RBS T7 from pET28, **<i>ATR_tr</i>** , T7 terminator from pET28)	This study
SbN_LX-pJ	KmR, pBBR1, Lv1 pSEVA23g19g1 vector containing pJ23100, RBS T7 from pET28, **<i>Sb6H_trN</i>** and **<i>ATR_tr</i>** genes fusion with	This study

	L_Mon2/L_GS linker, and T7 terminator from pET28	
SbN_LX-rhaBAD	KmR, pBBR1, Lv1 pSEVA23g19g1 vector containing RhaS gene, RhaBAD promoter, RBS T7 from pET28, <i>Sb6H_trN</i> and <i>ATR_tr</i> genes fusion with L_Mon1/L_Mon2/L_Hyd1/L_GS/L_L1/L_L2/L_WT linker, and T7 terminator from pET28	This study
SbNH_LX-rhaBAD	KmR, pBBR1, Lv1 pSEVA23g19g1 vector containing RhaS gene, RhaBAD promoter, RBS T7 from pET28, N-Histag, <i>Sb6H_trN</i> and <i>ATR_tr</i> genes fusion with L_Mon1/L_Mon2/L_Hyd1/L_GS/L_L1/L_L2/L_WT linker, and T7 terminator from pET28	This study

Supplementary Table S2. Plasmid maps of construct used in this study.

ID	Plazmid map
	<i>N-terminal library</i>
Sb-pJ	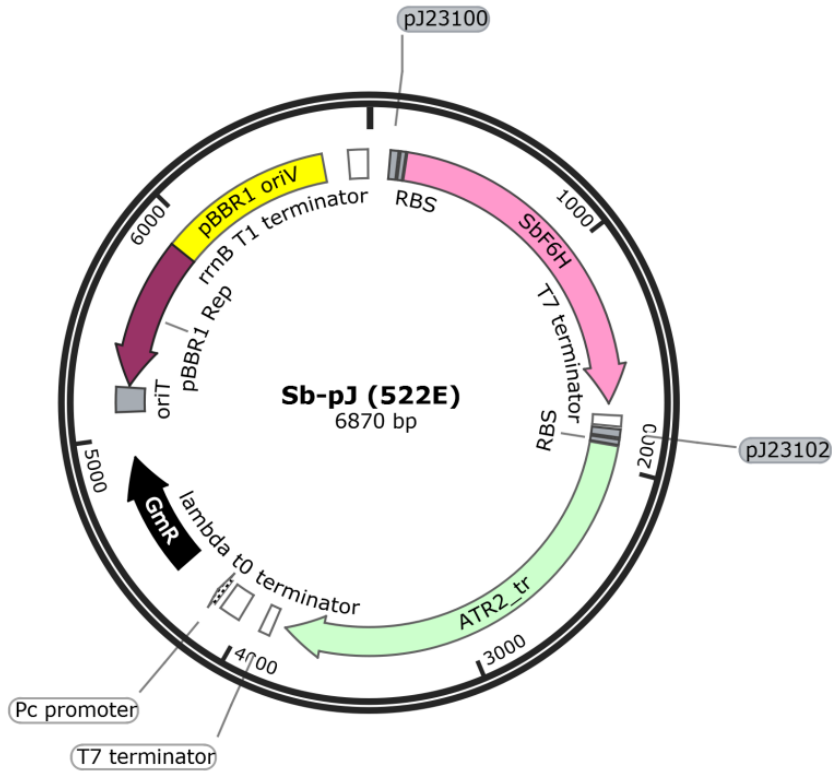 The diagram shows a circular plasmid map for Sb-pJ (522E), which is 6870 bp in size. The map is divided into several colored segments representing different genetic elements: a yellow segment for pBBR1 oriV (approx. 6000 bp), a pink segment for Sb6H (approx. 1000 bp), a green segment for ATR2_tr (approx. 3000 bp), and a black segment for GmR (approx. 4000 bp). Other elements include the pBBR1 Rep (approx. 1500 bp), the lambda to terminator (approx. 400 bp), the Pc promoter (approx. 1000 bp), and the T7 terminator (approx. 200 bp). The map also shows the RBS (Ribosome Binding Site) and the T7 terminator. The plasmid is labeled with pJ23100 and pJ23102. The map is titled 'Sb-pJ (522E) 6870 bp'.

SbN-pJ*

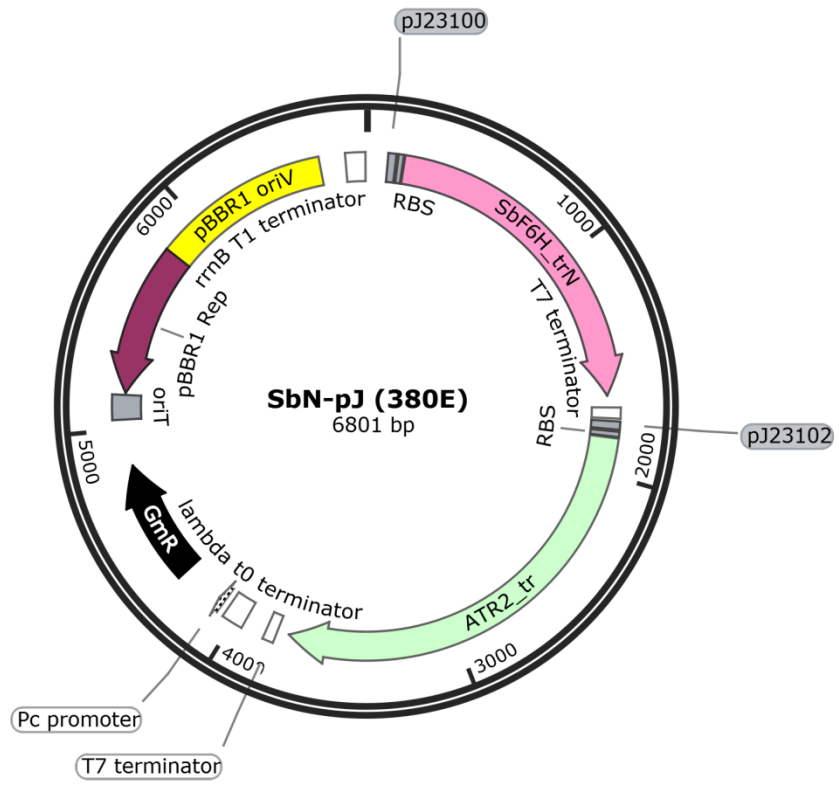

SbN-prhaB*

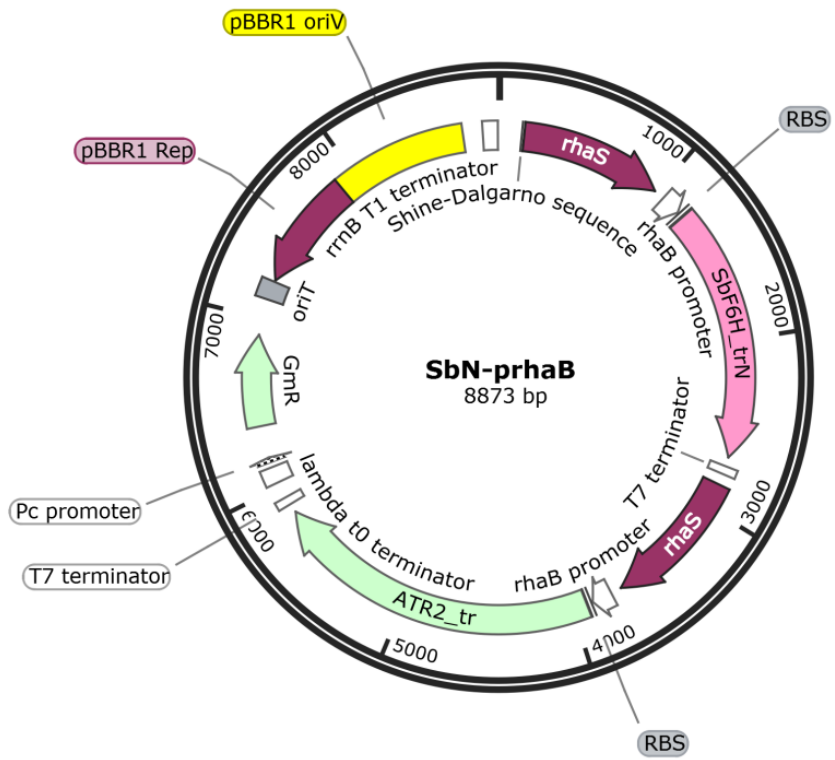

SbM-pJ

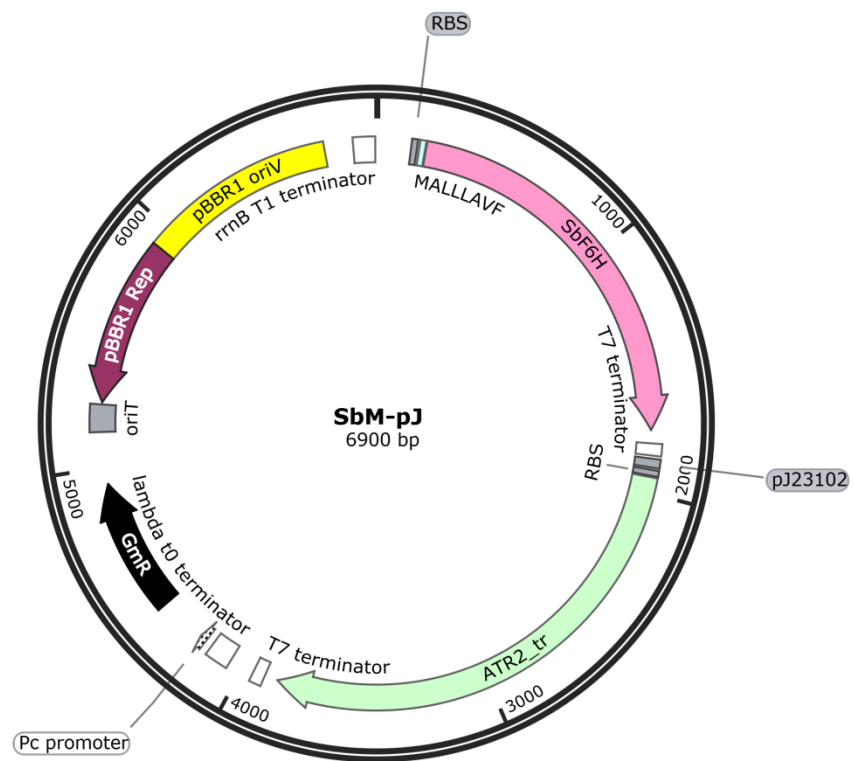

SbNM-pJ*

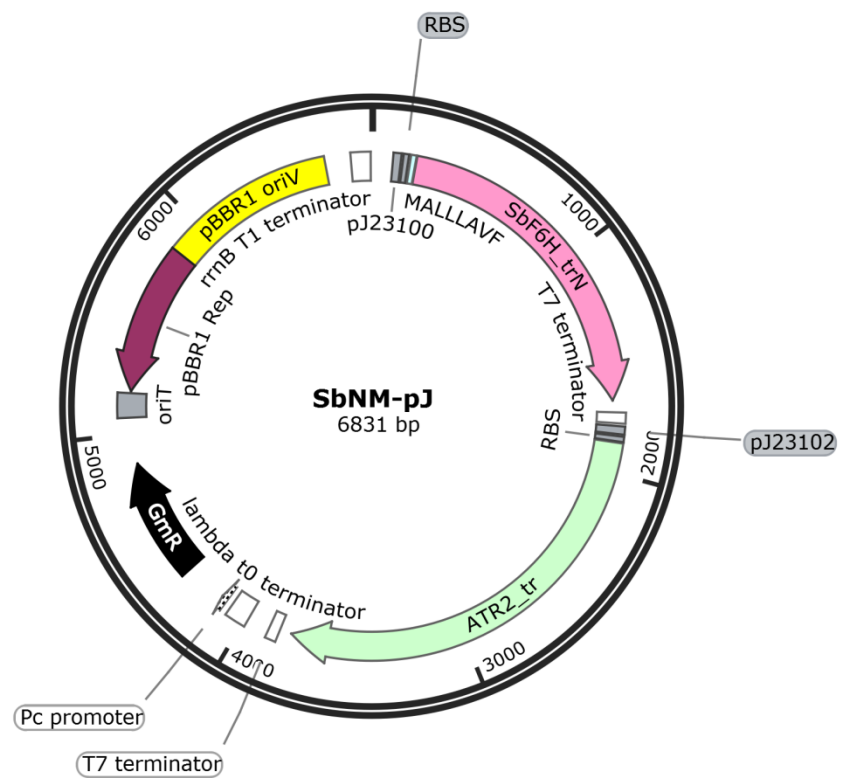

Gm-pJ

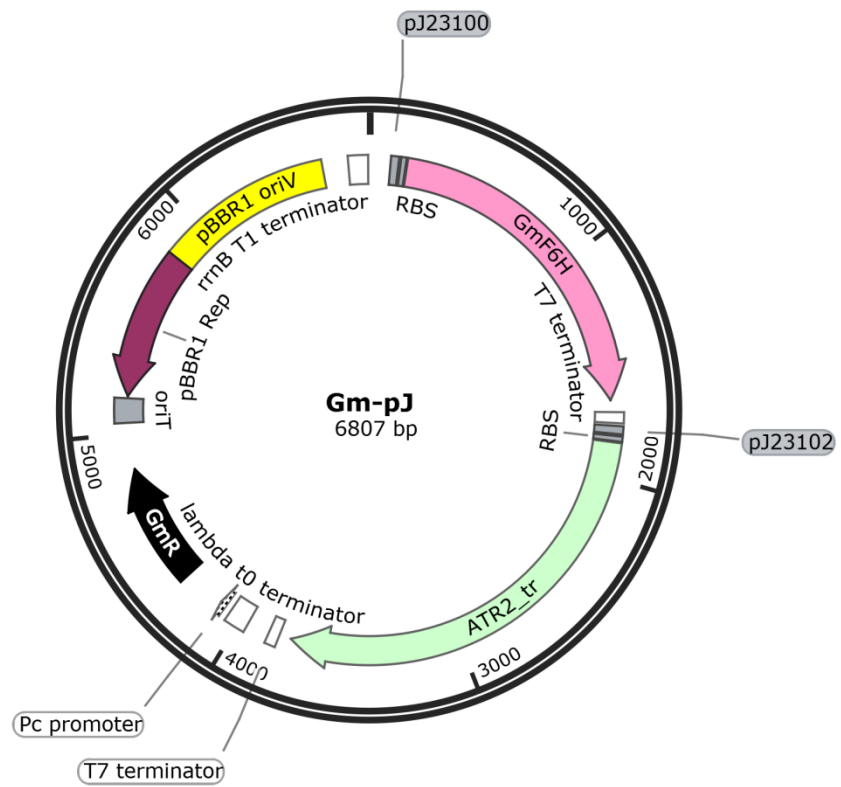

GmN-pJ*

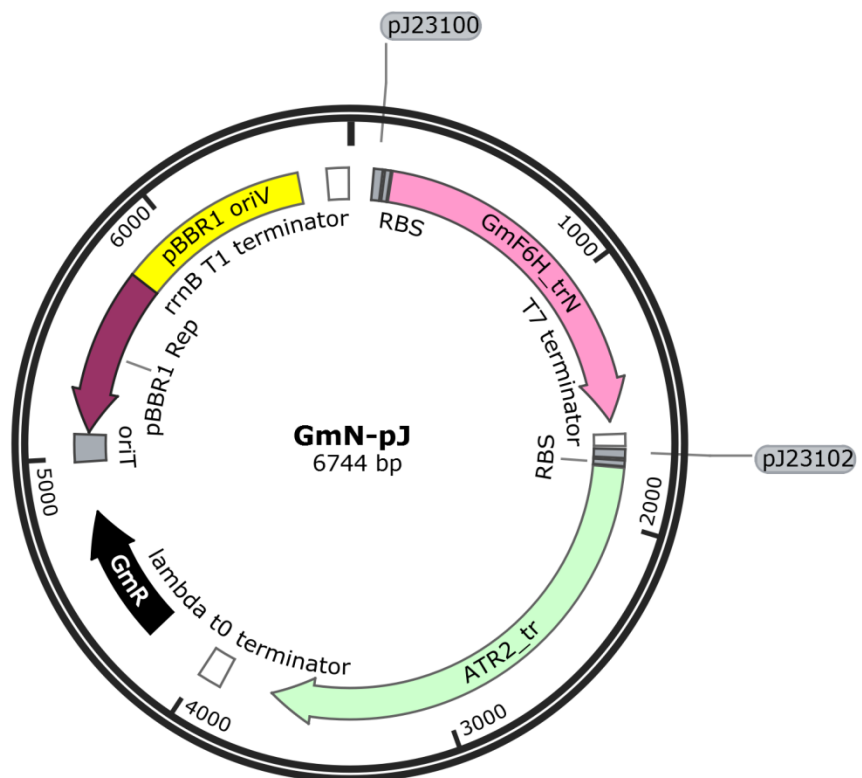

GmM-pJ

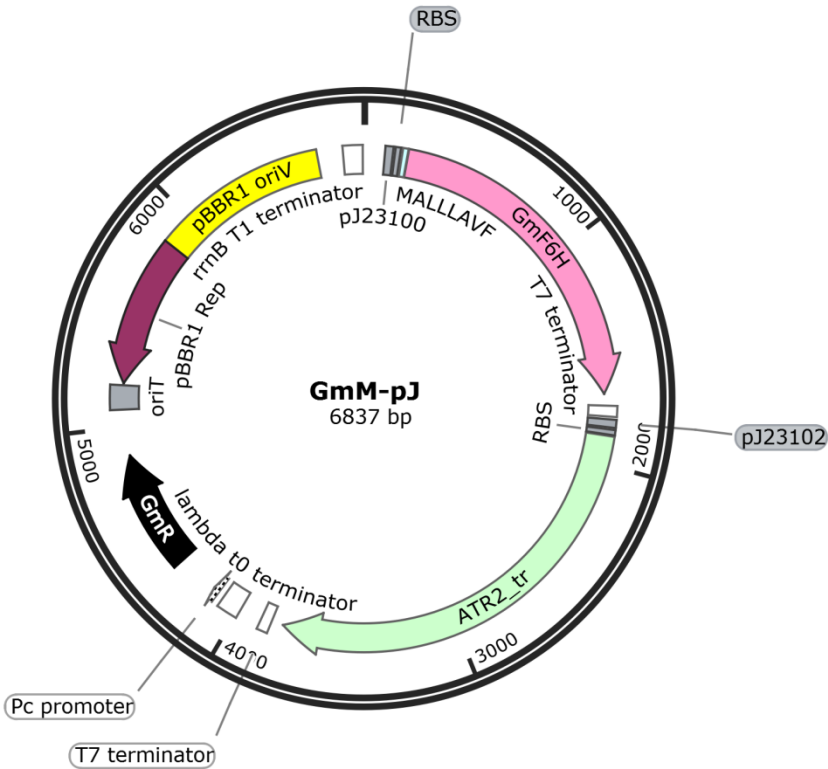

GmNM-pJ*

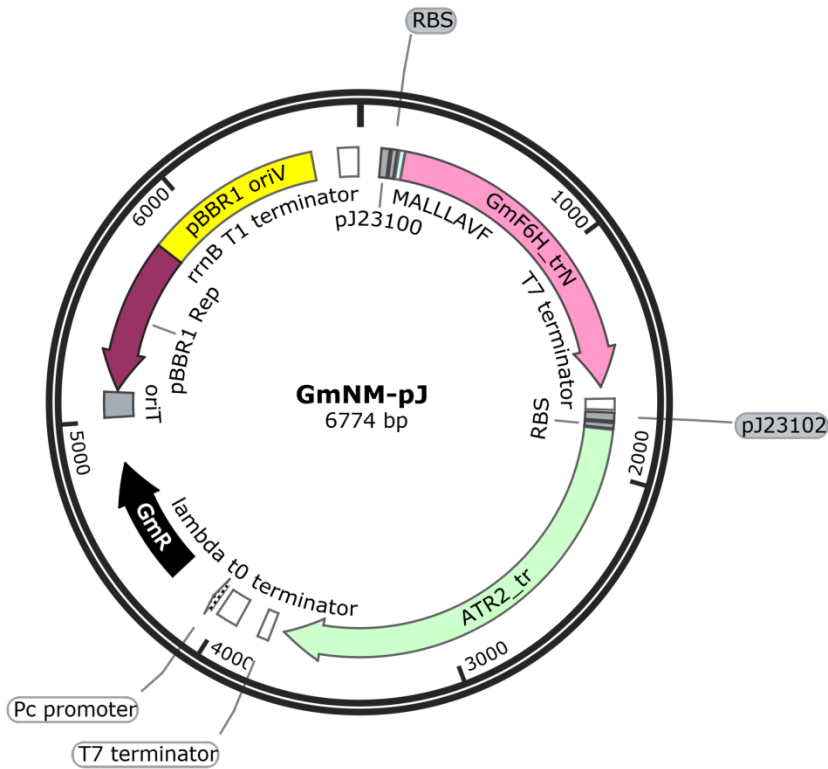

Fusion protein

SbN_LGS-pJ*

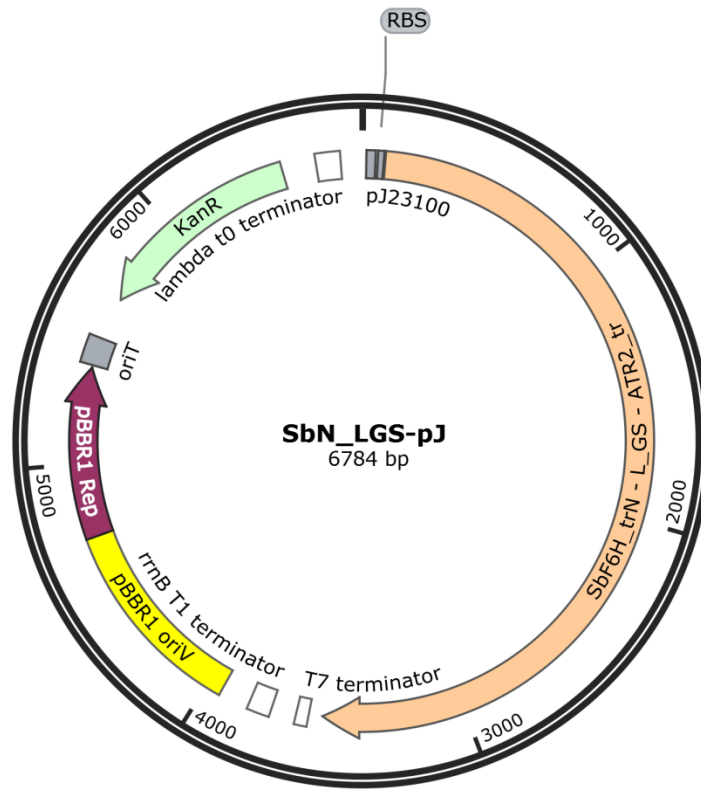

SbN_LMon2-pJ*

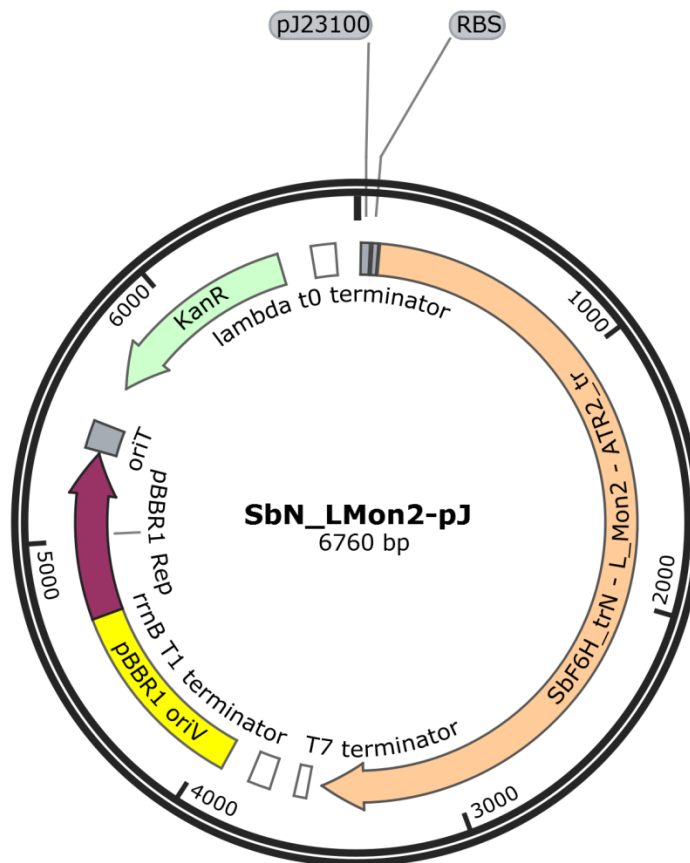

SbN_LGS-prhaB*

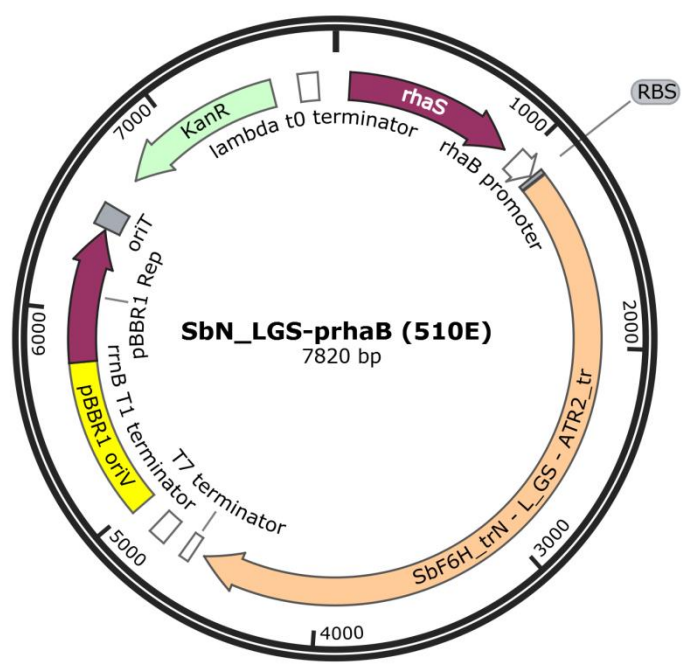

SbN_LL1-prhaB*

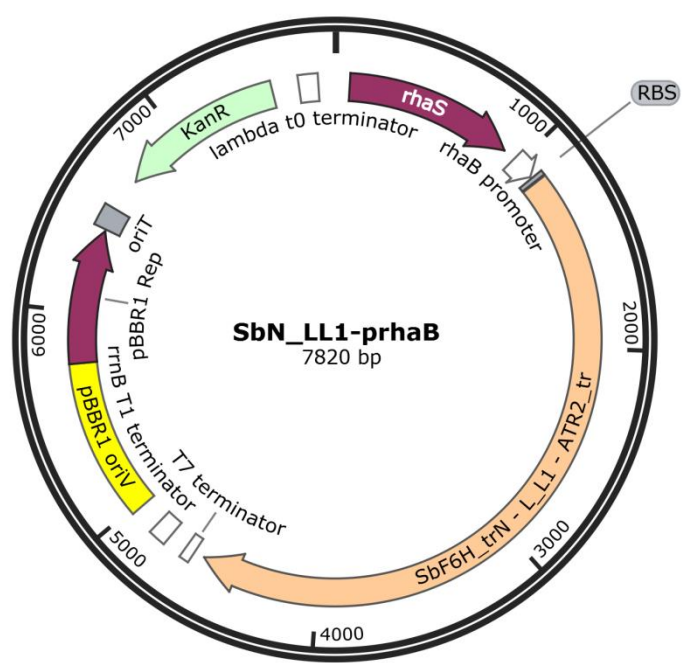

SbN_LL2-prhaB*

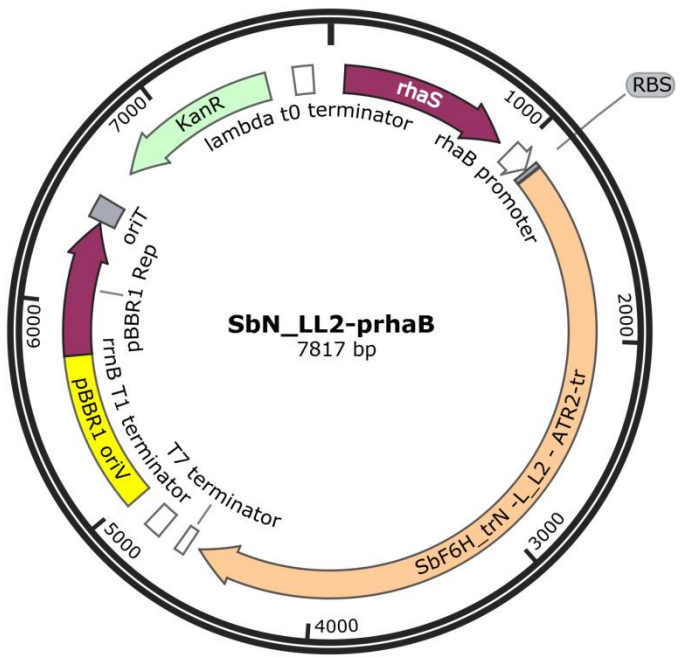

SbN_LWT-prhaB*

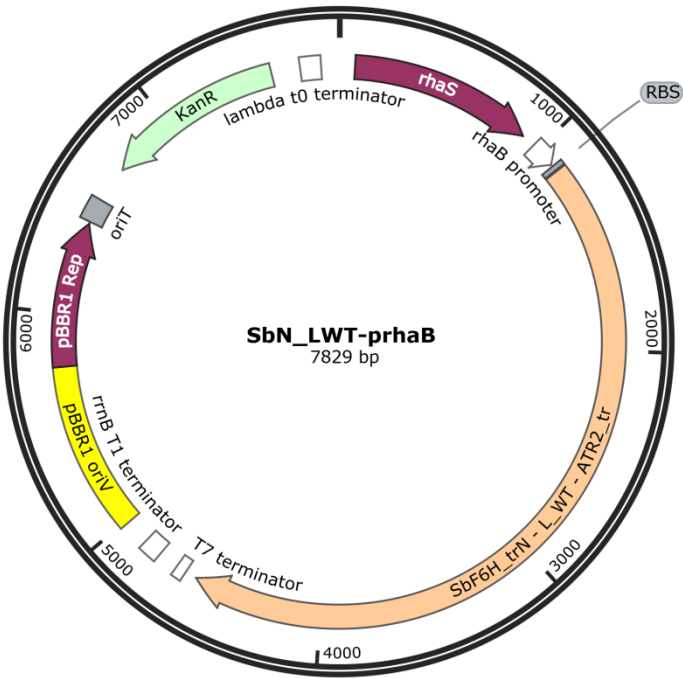

SbN_LMon1-
prhaB*

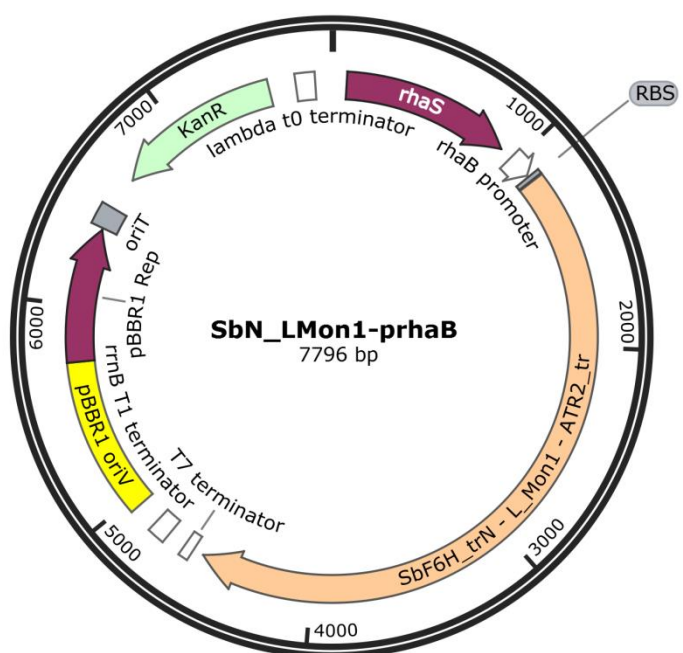

SbN_LMon2-
prhaB*

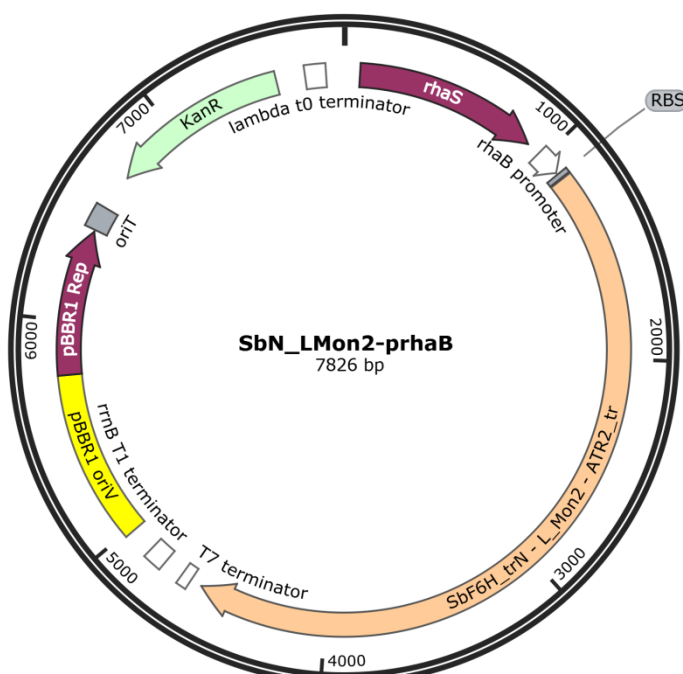

SbN_LHyd1-
prhaB*

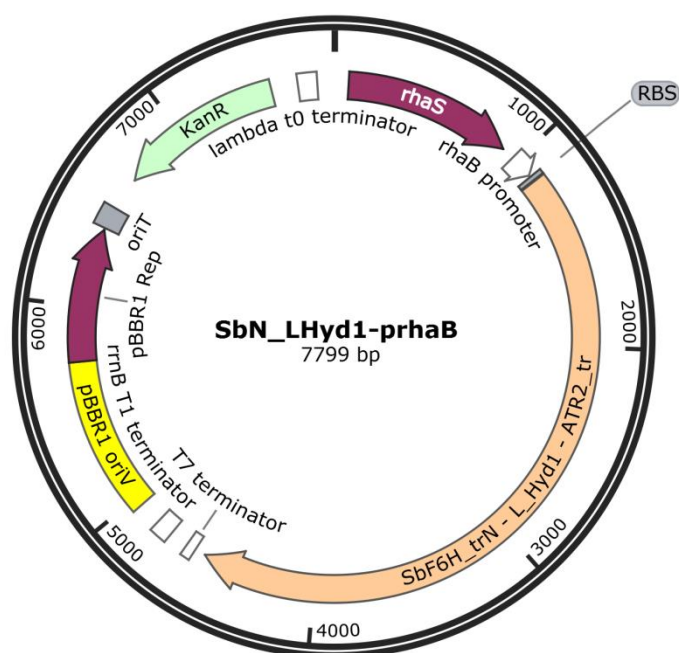

*trN – transmembrane anchor truncation

Supplementary Table S3. Structure of substrates used in this work. The shaded boxes in colour represent compounds transformed by Sb6H.

Name	Structure	Reaction results
Naringenin		+ (1)
Hesperetin		-
2'-hydroxyflavanone		-

3'-hydroxyflavanone

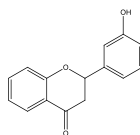

-

4'-hydroxyflavanone

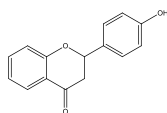

-

6-hydroxyflavanone

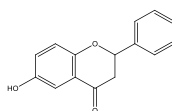

-

7-hydroxyflavanone

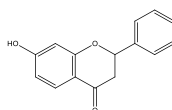

-

Isoxanthohumol

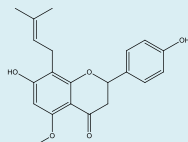

+ (1)

Eriodictyol

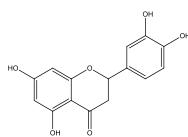

-

Chrysin

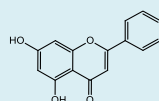

+ (1)

Diosmetin

-

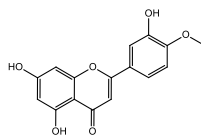

Apigenin

+ (1)

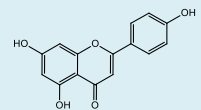

Luteolin

+(1)

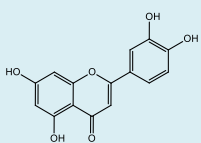

3-hydroxyflavone

-

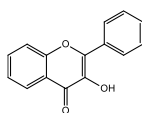

4',7-dihydroxyflavone

-

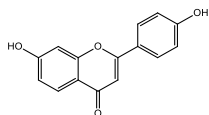

Quercetin

-

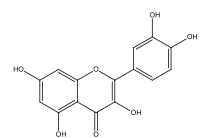

Fisetin

-

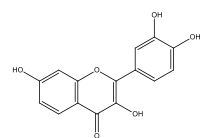

Kaempferol

+ (1)

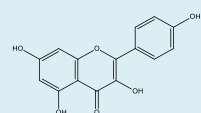

Biochanin A

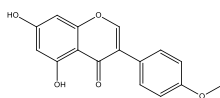

-

Daidzein

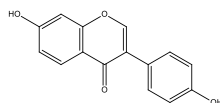

-

Genistein

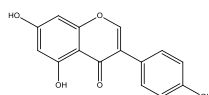

-

Phloretin

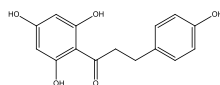

-

Resveratrol

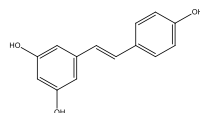

-

Xanthohumol

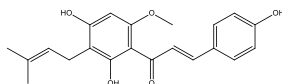

-

Supplementary Table S4. Results of one-way ANOVA with Levene's test for homogeneity of variances and Tukey's HSD post hoc analysis of cross-reactivity test assessing bacterial strains growth with and without supplementation after 18 h of cultivation. Inducer concentrations: 0.5 mM 5-ALA, 15 mM rhamnose. If needed, a Box-Cox transformation was performed to unify the variance of the data.

Expression system	F	P	Tukey's post hoc	SbN-pJ	SbN-pJ + 5-ALA	GmN-pJ	GmN-pJ + 5-ALA
constitutive	ANOVA	171.35	0.000	SbN-pJ	0.928	0.000	0.000
				SbN-pJ + 5-ALA	0.928	0.000	0.000
				GmN-pJ	0.000	0.000	0.737
	Levene's	1.635	0.233	GmN-pJ + 5-ALA	0.000	0.000	0.737
				SbN-LMon2-pJ	SbN-LMon2-pJ + 5-ALA	SbN-LGS-pJ	SbN-LGS-pJ + 5-ALA

constitutive	ANOVA	15.78	0.000	SbN-LMon2-pJ	0.000	0.051	0.008
				SbN_LMon2-pJ + 5-ALA	0.000	0.011	0.070
	Levene's	0.451	0.721	SbN_LGS-pJ	0.051	0.011	0.716
				SbN_LGS-pJ + 5-ALA	0.008	0.070	0.716
rhamnose-induced	ANOVA	4.26	0.050	SbN_LGS-prhaB	0.094	0.094	0.0623
				+ 5-ALA	0.094	0.961	0.961
	Levene's	0.750	0.500	+ 5-ALA, Rhm	0.063	0.961	0.961
rhamnose-induced	ANOVA	4.93	0.036	SbN_LMon2-prhaB	0.433	0.433	0.030
				+ 5-ALA	0.433	0.215	0.215
	Levene's	0.137	0.874	+ 5-ALA, Rhm	0.030	0.215	0.215

Supplementary Table S5. Results of one-way ANOVA with Levene's test for homogeneity of variances and Tukey's HSD post hoc analysis of cross-reactivity test assessing baicalein production by different N-terminal variants of SbF6H and GmF6H after 24 h of *in vivo* reaction. If needed, a Box-Cox transformation was performed to unify the variance of the data.

CYP		F	P	Tukey's post hoc	Sb-pJ	SbN-pJ	SbM-pJ	SbNM-pJ
SbF6H	ANOVA	7.904	0.009	Sb-pJ		0.043	0.963	0.521
				SbN-pJ	0.043		0.083	0.006
	Levene's	2.468	0.137	SbM-pJ	0.963	0.083		0.302
				SbNM-pJ	0.521	0.006	0.302	
GmF6H	ANOVA	72.25	0.000	Gm-pJ		0.000	0.000	0.000
				GmN-pJ	0.000		0.031	0.031
	Levene's	3.60	0.065	GmM-pJ	0.000	0.031		1.000
				GmNM-pJ	0.000	0.031	1.000	

Supplementary Table S6. Results of one-way ANOVA with Levene's test for homogeneity of variances and Tukey's HSD post hoc analysis of cross-reactivity test assessing baicalein production by two N-terminally modified SbF6H variants (SbN-pJ and SbNM-pJ) in different *E. coli* host strains after 24 h of *in vivo* reaction. If needed, a Box-Cox transformation was performed to unify the variance of the data.

N-variant		F	P	Tukey's post hoc	DH10beta	BL21 (DE3)	Rosetta2 (DE3) Lys	Arctic Express (DE3)
SbN-pJ	ANOVA	2.182	0.168	DH10beta		0.992	0.996	0.192
				BL21(DE3)	0.992		1.000	0.277
	Levene's	13.22	0.002	Rosetta2 (DE3) Lys	0.996	1.000		0.256

				Arctic Express<br>(DE3)	0.192	0.277	0.256		
					DH5alpha	DH10beta	BL21<br>(DE3)	Rosetta2<br>(DE3)<br>Lys	Arctic<br>Express<br>(DE3)
SbNM-pJ	ANOVA	80.81	0.000	DH5alpha		0.625	0.000	0.000	0.000
				DH10beta	0.624857		0.000	0.000	0.000
				BL21(DE3)	0.000	0.000		0.920	0.038
	Levene's	0.893	0.503	Rosetta2 (DE3)<br>Lys	0.000	0.000	0.920		0.133
				Arctic Express<br>(DE3)	0.000	0.000	0.038	0.133	

Supplementary Table S7. Results of one-way ANOVA with Levene's test for homogeneity of variances and Tukey's HSD post hoc analysis of cross-reactivity test assessing baicalein production by SbF6H_trN-ATR2_tr fusion proteins linked by different linkers after 24 h of *in vivo* reaction. If needed, a Box-Cox transformation was performed to unify the variance of the data.

	F	P	Tukey's post<br>hoc	L_GS	L_L1	L_L2	L_WT	L_Mon1	L_Mon2	L_Hyd1
ANOVA	0.43	0.847	L_GS		0.977	0.990	1.000	0.771	0.992	0.967
			L_L1	0.977		1.000	0.997	0.995	1.000	1.000
Levene's	4.20	0.013	L_L2	0.990	1.000		0.999	0.987	1.000	1.000
			L_WT	1.000	0.997	0.999		0.890	0.999	0.994
			L_Mon1	0.771	0.995	0.987	0.890		0.984	0.997
			L_Mon2	0.992	1.000	1.000	0.999	0.984		1.000
			L_Hyd1	0.967	1.000	1.000	0.994	0.997	1.000	

Codon optimized sequence

>SbF6H_trN

ATGAAACCGAAAAAAGCAGCCTGAACGCGCCGCCGGAAGCGGGCGGCGCGCTTTATTA
CCGGCCATCTGCATCTGATGGATGGCCGAGCGCGAGCGATAAACTGCCGCATATTAACCTG
GGCCTGCTGGCGGATCAGCATGGCCCGATTTTTACCATTTCGCCTGGGCGTGCATCGCGCGGTG
GTGGTGAGCAGCTGGGAACTGGCGAAAGAAATTTTTACCACCCATGATACCGCGGTGATGGC
CCGCCCCGCGCCTGATTGCGGATGATTATCTGAGCTATGATGGCGCGAGCCTGGGCTTTAGCCC
GTATGGCCCGTATTGGCGCGAAATTCGCAAACCTGGTGACCACCGAACTGCTGAGCGCGCGCC
GCATTGAACTGCAGCGCGCGACCCGCGTGCGCGAAATTACCCAGTTTACCGGCGAACTGTAT
AAACTGTGGGAAGAAAAAAGATGGCAGCGGCCGCGTGCTGGTGGATATGAAACAGTGGC
TGGGCAACCTGAGCCTGAACCTGGTGAGCCGCATGGTGGTGGGCAAACGCTTTTATGGCGGC
GATGATAGCGAAACCACCAAACGCTGGCGCGGCGTGATGCGCGAATTTTTTCAGCTGATTGG
CCAGTTTATTCCGGGCGATGGCCTGCCGTTTCTGCGCTGGCTGGATCTGGGCGGCTTTGAAAA
ACGCACCCGCGATACCGCGTATGAACTGGATAAAAATTATTGCGATGTGGCTGGCGGAATATC
GCAAACGCGAATATAGCGGCGATGATAAAGAACAGTGCTTTATGGCGCTGATGCTGAGCCTG
GTGCAGGCGAACCCGACCCTGCAGCTGCATTATGATGCGGATACCATTATTAAAGCGACCTG
CCAGGTGCTGATTAGCGCGGCGAGCGATACCACCACCGTGATTCTGATTTGGGTGATTAGCCT
GCTGCTGAACAACGCGGATGTGCTGAAAAAAGTGCAGGAAGAACTGGATGAACAGGTGGGC
CGCGAACGCCGCGTGGAAGAAAGCGATATTAGCAACCTGCCGTATCTGCAGGCGGTGGTGA
AAGAAACCATGCGCCTGTATCCGCCGCGCCGTTTTCGCGGCGTGCGCGCGTTTAGCGAAGAT
TGCACCGTGGGCGGCTATCATATTCAGAAAGGCACCTTTCTGATTGTGAACCTGTGGAAACTG
CATCGCGATCCGCGCGTGTTGGAGCGATGATGCGCTGGAATTTAAACCGCAGCGCTTTTTTGAT
AAAAAAGTGGAAGTGAAAGGCCAGGATTTTGAACCTGATGCCGTTTGGCGGCGGCCGCCGCAT
GTGCCCAGGCAGCAACCTGGGCATGCATATGGTGCATTTTGTGCTGGCGAACATTCTGCAGGC
GTTTGATATTACCACCGGCAGCACCGTGATATGACCGAAAGCGTGGGCCTGACCAACATGA
AAGCGACCCCGCTGGATGCGATTCTGACCCCGCGCCTGAGCCCGACCCTGTATTAA

>GmF6H_trN

ATGAAAATTGTGACCAAAAAAAGCAACAGCACCCCGAGCCTGCCGCCGGGCCCCGTGGAAAC
TGCCGATTATTGGCAACATGCATAACCTGGTGGGCAGCCCGCTGCCGCATCATCGCCTGCCG
GATCTGAGCGCGAAATATGGCAGCCTGATGCATCTGAAACTGGGCGAAGTGAGCACCATTGT
GGTGAGCAGCCCCGGAATATGCGAAAGAAGTGATGAAAACCCATGATCATATTTTTGCGAGCC
GCCCCGTATGTGCTGGCGGCGGAAATTATGGATTATGATTTTAAAGGCGTGCGCTTTACCCCGT
ATGGCGATTATTGGCGCCAGCTGCGCAAAATTTTTGCGCTGGAACTGCTGAGCAGCAAACGC
GTGCAGAGCTTTTACGCCGATTTCGCGAAGAAGTGCTGACCAGCTTTATTAACGCATGGCGAC
CATTGAAGGCAGCCAGGTGAACGTGACCAAAGAAGTGATTAGCACCGTGTTTACCATTACCG
CGCGCACCCGCGCTGGGCAGCAAAAGCCGCCATCATCAGAAACTGATTAGCGTGGTGACCGA
AGCGGCGAAAATTAGCGGCGGCTTTGATCTGGGCGATCTGTATCCGAGCGTGAAATTTCTGC
AGCATATGAGCGGCCTGAAACCGAAACTGGAAAAACTGCATCAGCAGGCGGATCAGATTAT
GCAGAACATTATTAACGAACATCGCGAAGCGAAAAGCAGCGCGACCGGCGATCAGGGCGA
AGAAGAAGTGCTGCTGGATGTGCTGCTGAAAAAAGAATTTGGCCTGAGCGATGAAAGCATT
AAGCGGTGATTTGGGATATTTTTGGCGGCGGCAGCGATACCAGCAGCGCGACCATTACCTGG
GCGATGGCGGAAATGATTAAAAACCCGCGCACCATGGAAAAAGTGACAGCCGAAGTGCGCC
GCGTGTTTGATAAAGAAGGCCGCCCGAACGGCAGCGGCACCGAAAAACCTGAAATATCTGAA
AAGCGTGCTGAGCGAAACCCTGCGCCTGCATCCGCCGGCGCCGCTGCTGCTGCCGCGCGAAT
GCGGCCAGGCGTGCGAAATTAACGGCTATCATATTCCGGCGAAAAGCCGCGTGATTGTGAAC
GCGTGGGCGATTGGCCGCGATCCGCGCCTGTGGACCGAAGCGGAACGCTTTTATCCGGAACG
CTTTATTGAACGCAGCATTGAATATAAAAGCAACAGCTTTGAATTTATTCCGTTTGGCGCGGG
CCGCCGCATGTGCCCAGGCCTGACCTTTGGCCTGAGCAACGTGGAATATGTGCTGGCGATGCT
GATGTATCATTTTTGATTGGAAACTGCCGAAAGGCACCAAAAACGAAGATCTGGGCATGACCG
AAATTTTTGGCATTACCGTGCGCGCAAAGATGATCTGTATCTGATTCCGAAAACCGTGCATA
ACTAA

Media composition:

Lysogeny Broth (LB): 10 g/L tryptone. 5 g/L yeast extract. 10 g/L NaCl. pH 7.0

Terrific Broth (TB): 12 g/L tryptone. 24 g/L yeast extract. 4 mL glycerol. 3.8 g/L KH₂PO₄. 12.5 g/L K₂HPO₄

Dynamite Medium (DM):): 12 g/L tryptone. 24 g/L yeast extract. 7.75 mL glycerol. 5 g/L glucose. 3.8 g/L KH₂PO₄. 12.5 g/L K₂HPO₄. 0.195 g/L MgSO₄.
